# Auxin-mediated RcARF8-RcWRKY29-RcERF98 module protects the rose petals against the *Botrytis cinerea* by modulating ascorbic acid biosynthesis and reactive oxygen species scavenging

**DOI:** 10.1093/hr/uhag224

**Published:** 2026-06-03

**Authors:** Jie Wu, Mengyue Jing, Shiqiu Yang, Qiushi Li, Daliang Li, Qi Fu, Xujun Ma, Dan Wang, Jiaxing Li, Hongying Jian, Huijun Yan, Kaixue Tang, Hao Zhang, Xianqin Qiu, Weikun Jing

**Affiliations:** Flower Research Institute of Yunnan Academy of Agricultural Sciences, Kunming, Yunnan 650205, China; Vegetables and Flowers Institute of Inner Mongolia Academy of Agricultural & Animal Husbandry Sciences, Hohhot 010031, China; Flower Research Institute of Yunnan Academy of Agricultural Sciences, Kunming, Yunnan 650205, China; Flower Research Institute of Yunnan Academy of Agricultural Sciences, Kunming, Yunnan 650205, China; Institute of Plant Resources, Yunnan University, Kunming, Yunnan 650091, China; Flower Research Institute of Yunnan Academy of Agricultural Sciences, Kunming, Yunnan 650205, China; Institute of Plant Resources, Yunnan University, Kunming, Yunnan 650091, China; Flower Research Institute of Yunnan Academy of Agricultural Sciences, Kunming, Yunnan 650205, China; Institute of Plant Resources, Yunnan University, Kunming, Yunnan 650091, China; Flower Research Institute of Yunnan Academy of Agricultural Sciences, Kunming, Yunnan 650205, China; Institute of Plant Resources, Yunnan University, Kunming, Yunnan 650091, China; Flower Research Institute of Yunnan Academy of Agricultural Sciences, Kunming, Yunnan 650205, China; Institute of Plant Resources, Yunnan University, Kunming, Yunnan 650091, China; Flower Research Institute of Yunnan Academy of Agricultural Sciences, Kunming, Yunnan 650205, China; Institute of Plant Resources, Yunnan University, Kunming, Yunnan 650091, China; Flower Research Institute of Yunnan Academy of Agricultural Sciences, Kunming, Yunnan 650205, China; Flower Research Institute of Yunnan Academy of Agricultural Sciences, Kunming, Yunnan 650205, China; Flower Research Institute of Yunnan Academy of Agricultural Sciences, Kunming, Yunnan 650205, China; Flower Research Institute of Yunnan Academy of Agricultural Sciences, Kunming, Yunnan 650205, China; Flower Research Institute of Yunnan Academy of Agricultural Sciences, Kunming, Yunnan 650205, China; Flower Research Institute of Yunnan Academy of Agricultural Sciences, Kunming, Yunnan 650205, China; Flower Research Institute of Yunnan Academy of Agricultural Sciences, Kunming, Yunnan 650205, China

## Abstract

As one of the world’s most important ornamental crops, rose (*Rosa sp.*) is highly susceptible to grey mold disease caused by *Botrytis cinerea*. Outbreaks of this disease during long-distance transportation often lead to substantial economic losses. This study identifies the WRKY transcription factor RcWRKY29 as a key regulator in the defense response against *B. cinerea*. Integrated RNA sequencing and DNA affinity purification sequencing analyses revealed *RcERF98* as a direct target of RcWRKY29. Knockdown or overexpression *RcWRKY29* correspondingly altered *RcERF98* transcript levels, which in turn modulated ascorbic acid (AsA) accumulation and reactive oxygen species (ROS) scavenging in petals. Furthermore, exogenous application of naphthalene acetic acid significantly enhanced disease resistance and concurrently upregulated *RcWRKY29* expression. We demonstrated that the auxin response factor RcARF8, but not RcARF1, binds to the *RcWRKY29* promoter and activates its transcription. Moreover, knocking down or overexpression of *RcARF8* affected the expression of both *RcWRKY29* and *RcERF98*, thereby regulating AsA biosynthesis and ROS homeostasis, and ultimately determining disease susceptibility. Collectively, our findings reveal a signaling module in which the auxin-mediated transcription cascade involving RcARF8-RcWRKY29-RcERF98 confers resistance to *B. cinerea* by coordinating AsA-dependent ROS scavenging in rose petals.

## Introduction

Cut rose (*Rosa* spp.), the most economically valuable floricultural crop worldwide, contributes to over 30% of the global yield and market value of cut flowers. However, its postharvest quality and commercial value are seriously threatened by *Botrytis cinerea,* a major fungal pathogen responsible for economic losses exceeding 35% during long-distance transport. Accumulating evidence indicates that plant immune responses to various pathogens are critically modulated by complex hormonal networks, including auxins, cytokinin (CK), gibberellins, and ethylene, salicylic acid (SA), brassinosteroids, and jasmonic acid (JA) [1–4].

Among these phytohormones, auxin predominantly features as a regulatory hub that interconnects plant growth pathways with immune signaling [5–9]. Auxin enhances plant disease resistance by modulating defense-related pathways and physiological processes. For example, moderate auxin levels confer resistance to *B. cinerea* in kiwifruit [10]. In tobacco, calmodulin-mediated binding to YUC proteins (YUC1, YUC3, YUC5, and YUC6) promotes IAA biosynthesis, which subsequently activates phytosulfokine-induced immunity against the same pathogen [11]. Auxin response factors (ARFs), the key transcriptional regulators of auxin signaling, display remarkable functional diversity in plant immunity. For example, in rice (*Oryza sativa*), viral proteins may interact with some ARFs to suppress immunity and enhance susceptibility [12], whereas other ARFs play active roles in promoting resistance; OsARF17 enhances antiviral defense through specific protein interactions [13], and in tomato (*Solanum lycopersicum*), StARF16 attenuates immunity to necrotrophic pathogens by directly binding to the *StNPR1* promoter [14]. Furthermore, knockdown *OsARF12* in rice results in heightened susceptibility to the fungal pathogen *Magnaporthe oryzae* [15]. Despite accumulating insights into ARF-mediated immunity across various plant systems, the specific roles and regulatory mechanisms of ARFs in modulating defense responses against *B. cinerea* in rose flowers remain largely unknown.

Integrating downstream of primary hormone signaling, WRKY transcription factors serve as pivotal executors of innate plant immunity. In *B. cinerea*-infected roses, multiple WRKY genes are differentially induced and exert a range of regulatory functions that can be diverse or even antagonistic. For example, loss of *RcWRKY13* function enhances resistance, whereas knocking out *RcWRKY41* compromises it [16, 17]. Notably, WRKY proteins frequently converge with auxin signals by physically or transcriptionally coupling with ARFs form hierarchical ARF-WRKY regulatory modules [18–21]. These modules act as universal intersecting hubs coordinating diverse environmental stimuli. For instance, operating strictly cooperatively, ARF5 and WRKY40 coactivate the IPT promoter to drive Agrobacterium-induced tumor formation [22], while in rice, OsARF12 directly targets the *OsWRKY13* promoter to boost antiviral immunity [23]. Analogous ARF-WRKY complexes also profoundly govern abiotic tolerances, such as frost and drought adaptation in apples [24, 25]^.^ Although such complexes undeniably orchestrate the critical balance between plant growth and systemic stress responses globally, whether an ARF-WRKY molecular cascade explicitly mediates innate resistance to *B. cinerea* in roses remain completely unexplored.

Reactive oxygen species (ROS) are central signaling molecules in plant immunity, and their dynamic homeostasis is crucial for effective defense against pathogen invasion [26–28]. Pathogen infection commonly triggers rapid ROS accumulation, particularly H₂O₂, which activates early immune responses but may also cause oxidative damage if not properly controlled [29, 30]. To maintain redox balance, plants employ both enzymatic and non-enzymatic antioxidant systems, including superoxide dismutase, glutathione peroxidase, ascorbate peroxidase, catalase, and ascorbic acid (AsA) [31]. Emerging evidence indicates that ERF transcription factors are important regulators of this process. For example, SlERF.F4 enhances disease resistance in tomato by promoting antioxidant capacity and phenylpropanoid metabolism [32], whereas AtERF98 and RcERF98 stimulate AsA biosynthesis through activation of *VTC1*, thereby enhancing ROS scavenging ability in Arabidopsis and rose [33, 34]. These findings suggest that ERF-mediated redox regulation is an important component of plant stress adaptation. However, whether specific ERF members contribute to resistance against *B. cinerea* through modulation of AsA accumulation and ROS homeostasis in rose petals remains unclear.

In this study, we identified *RcWRKY29* as a positive regulator of rose resistance to *B. cinerea* that directly activates *RcERF98* expression. Exogenous application of naphthalene acetic acid (NAA) enhanced this resistance, and further analyses showed that the auxin-responsive factor RcARF8 is induced by *B. cinerea* infection and positively regulates *RcWRKY29* expression. Together, these findings establish an RcARF8-RcWRKY29-RcERF98 transcriptional module that promotes rose petal resistance to *B. cinerea* by modulating downstream defense responses and ROS homeostasis.

## Results

### *RcWRKY29* contributes to resistance against *B. cinerea* in rose petals

To identify key regulators involved in defense responses, we analyzed our transcriptomic data and focused on differentially expressed WRKY transcription factor genes. Among these, *RchiOBHmChr7g0196571* exhibited the significantly upregulation upon *B. cinerea* infection, suggesting a potential role in modulating resistance (Fig. 1A and Supplementary Fig. S1A). Subcellular localization analysis revealed the presence of this molecule in the nucleus (Supplementary Fig. S1C). Phylogenetic reconstruction placed the *Rosa chinensis* gene *RchiOBHmChr7g0196571* in the same clade as *Arabidopsis* WRKY29 (AT4G23550.1); consequently, the corresponding protein was designated *RcWRKY29* (Supplementary Fig. S1B). Despite the established roles of many WRKY genes in defense against *B. cinerea*, *RcWRKY29* is still largely unknown. To verify the role of *RcWRKY29* in response to *B. cinerea*, we conducted a virus-induced gene silencing (VIGS) assay in rose petals to reduce the *RcWRKY29* level, as described previously [17]. The silencing of *RcWRKY29* resulted in a significantly larger lesion area and higher ion leakage rate than those of the TRV control petal discs (Fig. 1B–E), indicating that *RcWRKY29* silencing promoted rose flower susceptibility to *B. cinerea*. Moreover, we overexpressed *RcWRKY29* in rose petals as described previously [35]. In contrast, *RcWRKY29* overexpression led to a smaller lesion area and lower ion leakage rate than did the pSuper1300 control (Fig. 1F–I), demonstrating that *RcWRKY29* increased tolerance to *B. cinerea* in rose petals.

**Figure 1:**
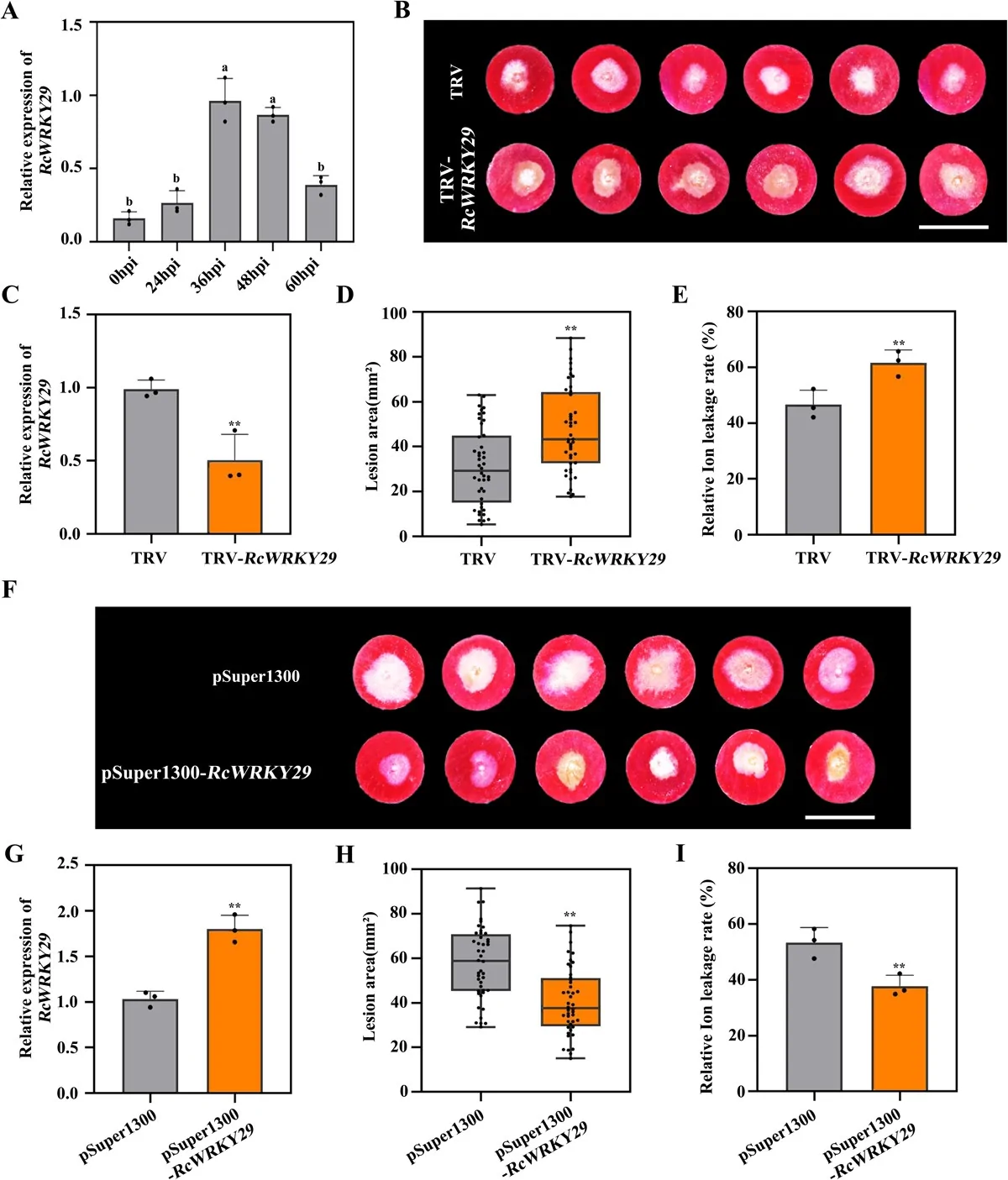
*RcWRKY29* positively regulates resistance to *B. cinerea* in rose petals. (A) Relative expression of *RcWRKY29* at different time points of infection (0, 24, 36, 48, 60 hpi). Values represent mean ± SD (*n* = 3). (B) Lesion phenotypes of petal discs in TRV-*RcWRKY29* and TRV control. White scale bar = 1 cm. (C–E) Relative expression of *RcWRKY29* (C) (*n* = 3), lesion area (D) (*n* = 50) and relative ion leakage rate (E) (*n* = 3) of petal discs in TRV-*RcWRKY29* and TRV. Values represent mean ± SD. (F) Lesion phenotypes of petal discs in pSuper1300-*RcWRKY29* and pSuper1300 control. White scale bar = 1 cm. (G–I) Relative expression of *RcWRKY29* (G) (*n* = 3), lesion area (H) (*n* = 50) and relative ion leakage rate (I) (*n* = 3) of petals in pSuper1300-*RcWRKY29* and pSuper1300. Values represent mean ± SD. Different lowercase letters (in A) indicate significant differences according to one-way ANOVA with Duncan’s multiple comparisons test (*P* < 0.05). Statistical significance was determined by Student’s *t*-test (^**^  *P* < 0.01).

### RcWRKY29 binds to the *RcERF98* promoter and activates its transcriptional activity, thereby modulating AsA biosynthesis and ROS scavenging

To verify the downstream regulatory mechanism of RcWRKY29, we conducted DNA affinity purification sequencing (DAP-seq) to screen the targets involved in the response to *B. cinerea*. By performing a joint analysis between the differentially expressed genes (DEGs) and the DAP-seq database, we identified 2824 DEGs and 9671 candidate *RcWRKY29* target genes (Fig. 2A and B). Subsequent Venn diagram analysis revealed 769 overlapping genes, suggesting their potential role as direct targets of *RcWRKY29* (Fig. 2C). Moreover, the Gene Ontology (GO) enrichment results revealed that these genes were enriched mainly in ‘response to chitin’, ‘response to nitrogen compound’, ‘response to stimulus’, and ‘response to hormone’ (Fig. 2D, Supplementary Table S1). Chitin has been widely reported to play a key role in defense against fungal pathogens. However, whether *RcWRKY29* can directly regulate these genes is still largely unknown. Hence, we performed a heatmap analysis of the 23 genes enriched in the ‘response to chitin’ category. Among them, the *RchiOBHmChr6g0288241*, *RchiOBHmChr2g0093571*, *RchiOBHmChr3g0476021*, and *RchiOBHmChr4g0442301* levels were significantly increased at 36 h after *B. cinerea* infection (Fig. 2E). A yeast one-hybrid (Y1H) assay was subsequently used to identify direct interactions. After verification, we found that RcWRKY29 interacted with the *RchiOBHmChr6g0288241* promoter but not with the other promoters (Fig. 3A, Supplementary Fig. S2). Phylogenetic analysis and conserved domain alignment revealed that *RchiOBHmChr6g0288241* is an AP2-ERF transcription factor, a homolog of *Arabidopsis* ERF98 (AT3G23230.1); hence, we named it *RcERF98* (Supplementary Fig. S3A). Subcellular localization analysis suggested that this molecule localizes to the nucleus (Supplementary Fig. S3B), and its expression was significantly increased after *B. cinerea* infection (Supplementary Fig. S3C). Moreover, chromatin immunoprecipitation PCR (ChIP-qPCR) and dual luciferase assays further demonstrated that RcWRKY29 could bind to the P5 region of the *RcERF98* promoter and increase its promoter activity (Fig. 3B–E). RT-qPCR then showed that the silencing or overexpression of *RcWRKY29* caused decreased or increased *RcERF98* expression, respectively (Fig. 3F and G). Moreover, silencing *RcERF98* consequently enhanced disease susceptibility in petal discs, as evidenced by significantly increased lesion area and ion leakage compared to the control (Fig. 4A–D), a phenotype consistent with *RcWRKY29* knockdown. In addition, *RcERF98* overexpression resulted in a smaller lesion area and a lower ion leakage rate than the pSuper1300 control (Fig. 4E–H), thereby mimicking the effects of *RcERF98* overexpression on *B. cinerea*. These results showed that compared to the pSuper1300 control, overexpression of *RcERF98* in rose petals demonstrated increased tolerance to *B. cinerea.* Based on our previous study, we have demonstrated that RcERF98 promotes salt stress resilience in roses by inducing the biosynthesis of AsA and the scavenging of ROS [33]. Moreover, ChIP-qPCR and dual luciferase assays confirmed that RcERF98 binds directly to the P2 region of the *RcVTC1* promoter and activates its promoter activity, which was the AsA synthesis gene (Fig. 5A and B) [32]. Overexpression or silencing of *RcERF98* and *RcWRKY29* led to corresponding changes in *RcVTC1* expression (Fig. 5C and E). Hence, we investigated the AsA content and ROS accumulation after *B. cinerea* infection. We found that overexpression of *RcWRKY29* and *RcERF98* significantly enhanced AsA biosynthesis and ROS scavenging compared to the pSuper1300 control (Fig. 5H and I). DAB staining revealed decreased staining intensity (Fig. 5G), and the expression of genes involved in these pathways exhibited similar upregulation (Supplementary Fig. S4E and F). Conversely, silencing of *RcWRKY29* and *RcERF98* resulted in decreasing AsA levels and H_2_O_2_ accumulation compared to the TRV control (Fig. 5K and L). DAB staining revealed significantly increased staining intensity (Fig. 5J), accompanied by downregulation of the corresponding biosynthetic and scavenging genes (Supplementary Fig. S4B and C). Together, our findings suggest that the RcWRKY29-RcERF98 module functions vitally in plant defense against *B. cinerea* by modulating the AsA biosynthesis and ROS scavenging.

**Figure 2:**
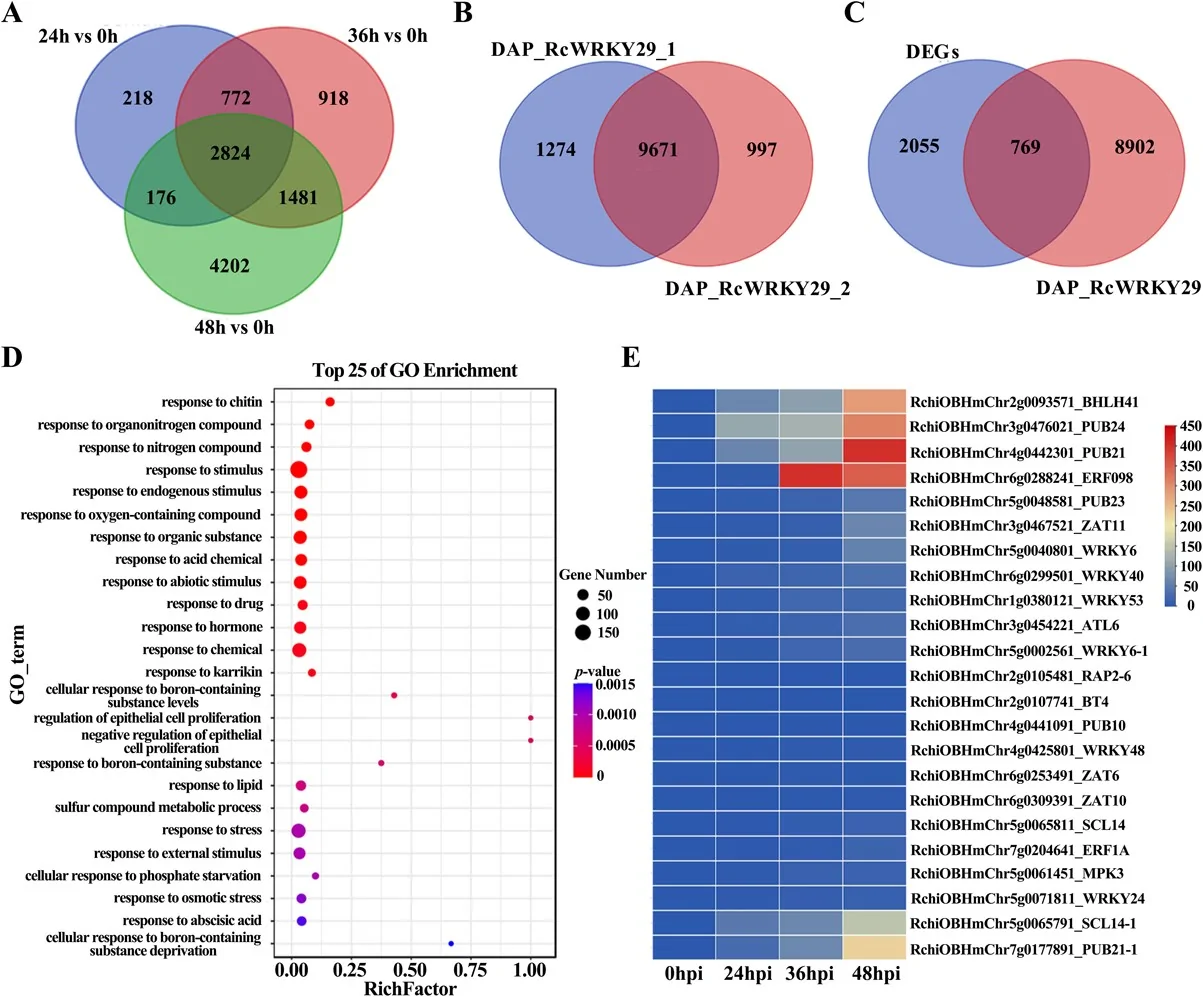
Screening of *RcWRKY29*-targeted genes via integrated analysis of transcriptome and DAP-seq data. (A) Venn diagram showing the overlap of DEGs across different time points (24, 36, 48 hpi) compared to the 0 hpi. (B) Venn diagram showing overlaps between *RcWRKY29*-binding regions (DAP_*RcWRKY29*_1 and DAP_*RcWRKY29*_2). (C) Overlap of DEGs and *RcWRKY29*-target genes (DAP_*RcWRKY29*). (D) GO enrichment analysis for the above overlapped genes. (E) Heatmap showing the expression level of *RcWRKY29* downstream genes across the selected time points (0, 24, 36, 48 hpi)

**Figure 3:**
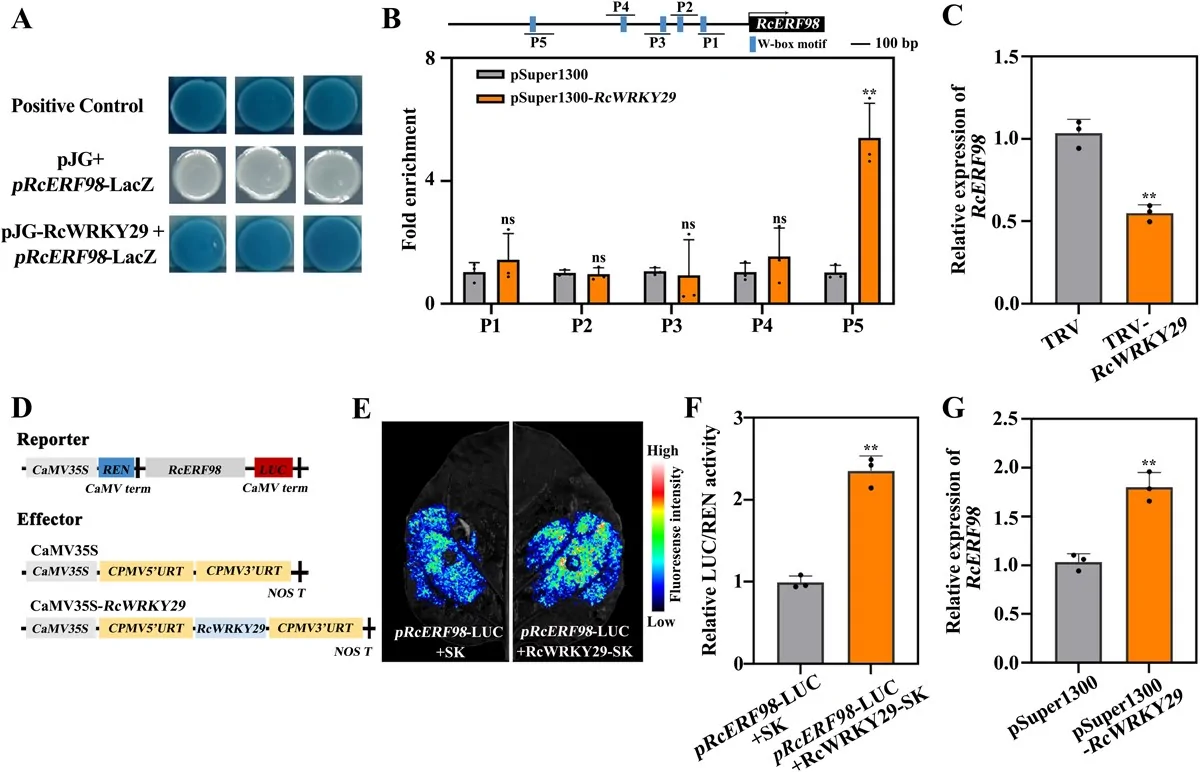
*RcERF98* was the target of RcWRKY29. (A) Y1H assay verified the binding of RcWRKY29 to the *RcERF98* promoter. (B) Chromatin immunoprecipitation (ChIP)–qPCR analysis of RcWRKY29 at different regions (P1–P5) on the *RcERF98* promoter. Values represent mean ± SD (*n* = 3). (C) Relative expression of *RcERF98* after *RcWRKY29* silencing. Values represent mean ± SD (*n* = 3). Diagrammatic representation of the effector and reporter cassettes used in trans-activation assays. Luciferase imaging of *N. benthamiana* leaves. (F) Quantitative analysis of the relative LUC/REN activity. Values represent mean ± SD (*n* = 3). G. Relative expression of *RcERF98* after *RcWRKY29* overexpression. Values represent mean ± SD (*n* = 3). Significance was evaluated by two-tailed Student’s *t*-test (^**^  *P* < 0.01, ns, not significant).

**Figure 4:**
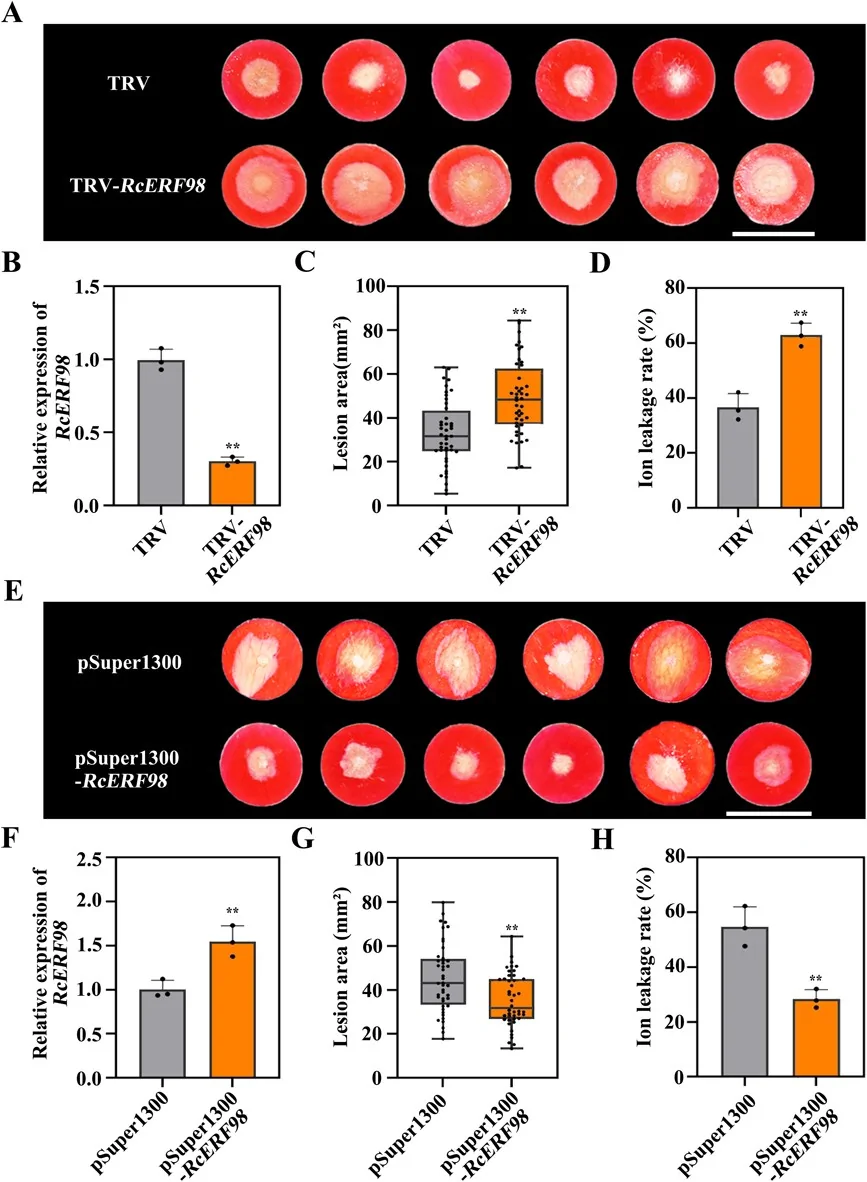
*RcERF98* positively regulates resistance to *B. cinerea* in rose petals. (A) Lesion phenotypes of petal discs in TRV-*RcERF98* and TRV control. White scale bar = 1 cm. (B–D) Relative expression of *RcERF98* (B) (*n* = 3), lesion area (C) (*n* = 50) and relative ion leakage rate (D) (*n* = 3) from TRV-*RcERF98* and TRV control petals. Values represent mean ± SD. (E) Lesion phenotypes of petal discs in pSuper1300-*RcERF98* and pSuper1300 control. White scale bar = 1 cm. (F–H) Relative expression of *RcERF98* (F) (*n* = 3), lesion area (G) (*n* = 50) and relative ion leakage rate (H) (*n* = 3) of petals in pSuper1300-*RcERF98* and pSuper1300 control. Values represent mean ± SD. Significance was evaluated by two-tailed Student’s *t*-test (^**^  *P* < 0.01).

**Figure 5:**
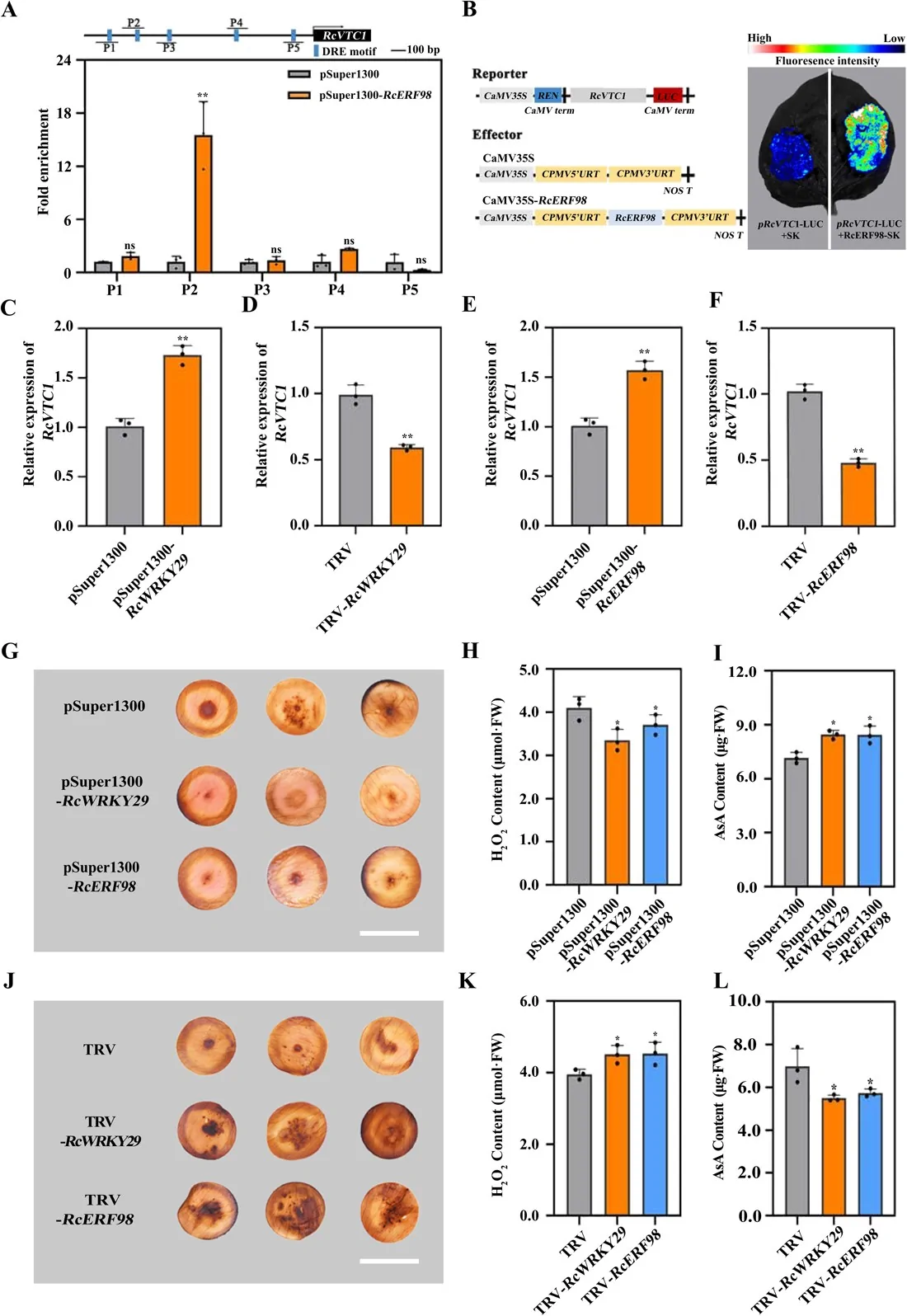
RcWRKY29-RcERF98 module regulates H_2_O_2_ and AsA levels by controlling *RcVTC1* expression. (A) ChIP-qPCR analysis of RcERF98 at different region (P1-P5) on the *RcVTC1* promoter. Values represent mean ± SD (*n* = 3). White scale bar = 1 cm. (B) Diagrammatic overview of the assembled reporter and effector cassettes, alongside representative bioluminescence images recorded following agro-infiltration of *N. benthamiana* leaves. (C–F) Relative expression of *RcVTC1* from pSuper1300-*RcWRKY29* (C), TRV-*RcWRKY29* (D)*,* pSuper1300-*RcERF98* (E), TRV-*RcERF98* (F)*,* and control petals. Values represent mean ± SD (*n* = 3). (G) DAB staining of pSuper1300-*RcWRKY29*, pSuper1300-*RcERF98* and pSuper1300 control of rose petals. White scale bar = 1 cm. (H and I) H_2_O_2_ and AsA levels in pSuper1300-*RcWRKY29*, pSuper1300-*RcERF98* and pSuper1300 control petals (*n* = 3). (J) DAB staining of TRV-*RcWRKY29*, TRV-*RcERF98* and TRV control of rose petals. White scale bar = 1 cm. (K and L) H_2_O_2_ and AsA levels in TRV-*RcWRKY29*, TRV-*RcERF98* and TRV petals (*n* = 3). Significance was evaluated by two-tailed Student’s *t*-test (^*^  *P* < 0.05, ^**^  *P* < 0.01, ns, not significant).

### Auxin-mediated RcARF8 positively regulates the RcWRKY29-RcERF98 module in response to *B. cinerea*

As widely reported, CK, ABA, JA and auxin play critical roles in defense signaling against infection by *B. cinerea* [17, 36]. Whether hormones influence *RcWRKY29* expression is still largely unknown. With respect to hormone treatment, NAA (50 μM) treatment significantly induced *RcWRKY29* expression at 36 hours postinoculation (hpi), but we found no significant differences under the 6-BA, ABA, or MeJA treatments (Fig. 6A). To explore the roles of auxin in response to *B. cinerea*, we quantified the relative IAA content in rose petals after *B. cinerea* infection and found that the IAA content significantly increased at 36 and 48 hpi (Fig. 6B). We further found that NAA treatment significantly enhanced disease resistance against *B. cinerea* by enhancing AsA biosynthesis and ROS scavenging (Fig. 6C–G). In contrast, NPA treatment impairs AsA synthesis and ROS scavenging capacity, thereby making petals more susceptible to disease (Fig. 6H–L). DAB staining showed that NAA treatment resulted in lighter staining in petals, whereas NPA treatment led to darker staining (Fig. 6E and J). Therefore, we hypothesized that auxin might modulate resistance against *B. cinerea* via *RcWRKY29*. The functions of auxin usually depend on ARFs [37–39]. Using the JASPAR CORE website (https://jaspar.genereg.net/), we predicted cis-elements in the *RcWRKY29* promoter and identified 8 auxin-responsive cis-elements (Fig. 7B). Among the 12 previously identified *ARF* genes, 6 presented high expression levels in petals [40]. Thus, we cloned the 6 ARF coding sequences (CDS) and conducted Y1H to identify the interaction between the 6 ARF proteins and *RcWRKY29*. Notably, RcARF1 and RcARF8 could interact with the *RcWRKY29* promoter in yeast, but the other genes could not (Fig. 7A, Supplementary Fig. S5A). Moreover, the expression of *RcARF1* and *RcARF8* was induced by NAA (Supplementary Fig. S5B and C). The dual luciferase assay results suggested that RcARF8, but not RcARF1, could bind to and activate the *RcWRKY29* promoter (Fig. 7C–E, Supplementary Fig. S5D–F). No significant change in resistance to *B. cinerea* was observed following the silencing of *RcARF1*, compared to the TRV control plants. ChIP-qPCR defined the P3 and P5 regions of the *RcWRKY29* promoter as direct targets of RcARF8 binding (Fig. 7B). Moreover, compared with the control, *RcARF8* knockdown resulted in a significantly larger lesion area and higher ion leakage rate (Fig. 8A–D), mimicking the results of *RcWRKY29* silencing, resulting in increased flower susceptibility to *B. cinerea*. Moreover, RT-qPCR suggested that the silencing of *RcARF8* caused decreased *RcWRKY29* and *RcERF98* expression (Fig. 8E and F). Silencing of *RcARF8* resulted in decreased AsA biosynthesis and ROS scavenging and resulted in deeper DAB staining of the petals (Fig. 8G–I and Supplementary Fig. S4A). Furthermore, compared with the pSuper1300 control, the overexpression of *RcARF8* in rose petals significantly promoted *B. cinerea* tolerance by regulating *RcWRKY29* and *RcERF98* expression (Fig. 8J–O). Besides, overexpression of *RcARF8* resulted in enhanced AsA biosynthesis and ROS scavenging, as well as a reduction in DAB staining intensity.(Fig. 8P–R and Supplementary Fig. S4D). In addition, *RcARF1* silencing did not affect petal resistance against *B. cinerea* (Supplementary Fig. S6A–D). We treated the samples with NPA, an auxin transport inhibitor, compared to the control (0 μM NPA), NPA(30 μM) treatment significantly reduced the expression levels of *RcWRKY29*, *RcERF98*, and *RcARF8* (Supplementary Fig. S7). These results suggest that endogenous auxin signaling may be required for activating these TFs and the downstream regulatory module. To further investigate the potential interactions among these transcription factors, we performed BiFC and yeast two-hybrid assays. The results revealed no direct protein–protein interactions between any two of RcWRKY29, RcERF98, and RcARF8 (Supplementary Fig. S8). In summary, we hypothesized that auxin may function through RcARF8-RcWRKY29-RcERF98 module to regulate resistance to *B. cinerea* infection in rose petals by modulating the AsA biosynthesis and ROS scavenging (Fig. 9).

**Figure 6:**
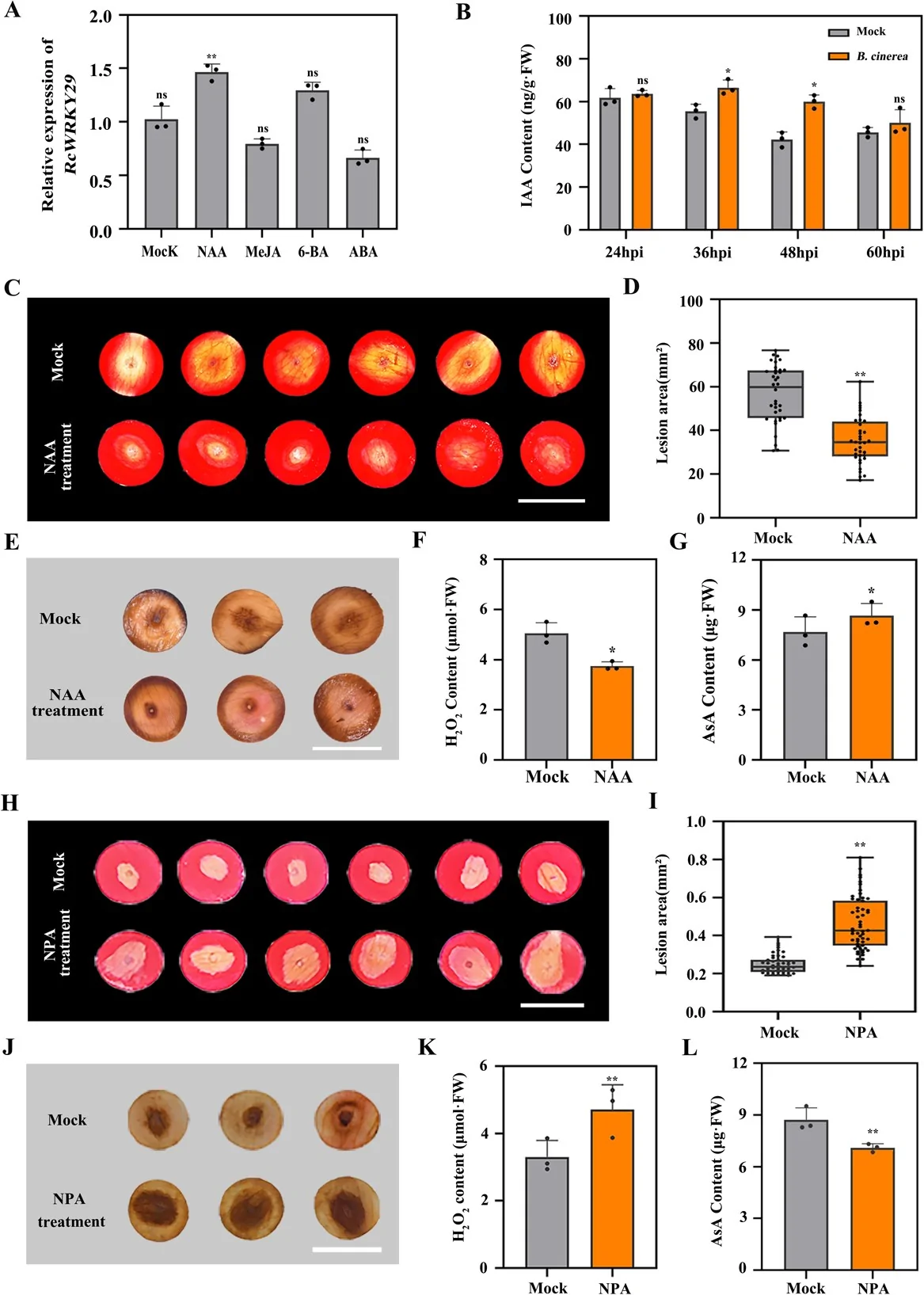
NAA and NPA treatments significantly affected the resistance of rose petals to *B. cinerea.* (A) Transcript abundance of *RcWRKY29* following exposure to Mock, NAA, MeJA, 6-BA, or ABA treatments. Values represent mean ± SD (*n* = 3). (B) Changes in IAA content during *B. cinerea* infection at 24, 36, 48, and 60 hpi under mock and pathogen-treated conditions (*n* = 3). (C) Representative images of lesion development on rose petals under mock and NAA treatment following infection by *B. cinerea*. White scale bar = 1 cm. (D) Lesion area of petal discs after NAA treatment. Values represent mean ± SD (*n* = 50). (E) DAB staining of NAA-treated petals compared to the control. White scale bar = 1 cm. (F and G) H_2_O_2_ and AsA levels of NAA treatment and control treatment petals(*n* = 3). (H) Representative images of lesion development on rose petals under mock and NPA treatment following infection by *B. cinerea*. White scale bar = 1 cm. (I) Lesion area of petal discs in mock and NPA. Values represent mean ± SD (*n* = 50). J. DAB staining of rose petals under mock and NPA. White scale bar = 1 cm. (K and L) H_2_O_2_ and AsA levels in petals of control and NPA (*n* = 3). Significance was evaluated by two-tailed Student’s *t*-test (^*^  *P* < 0.05, ^**^  *P* < 0.01, ns, not significant).

**Figure 7:**
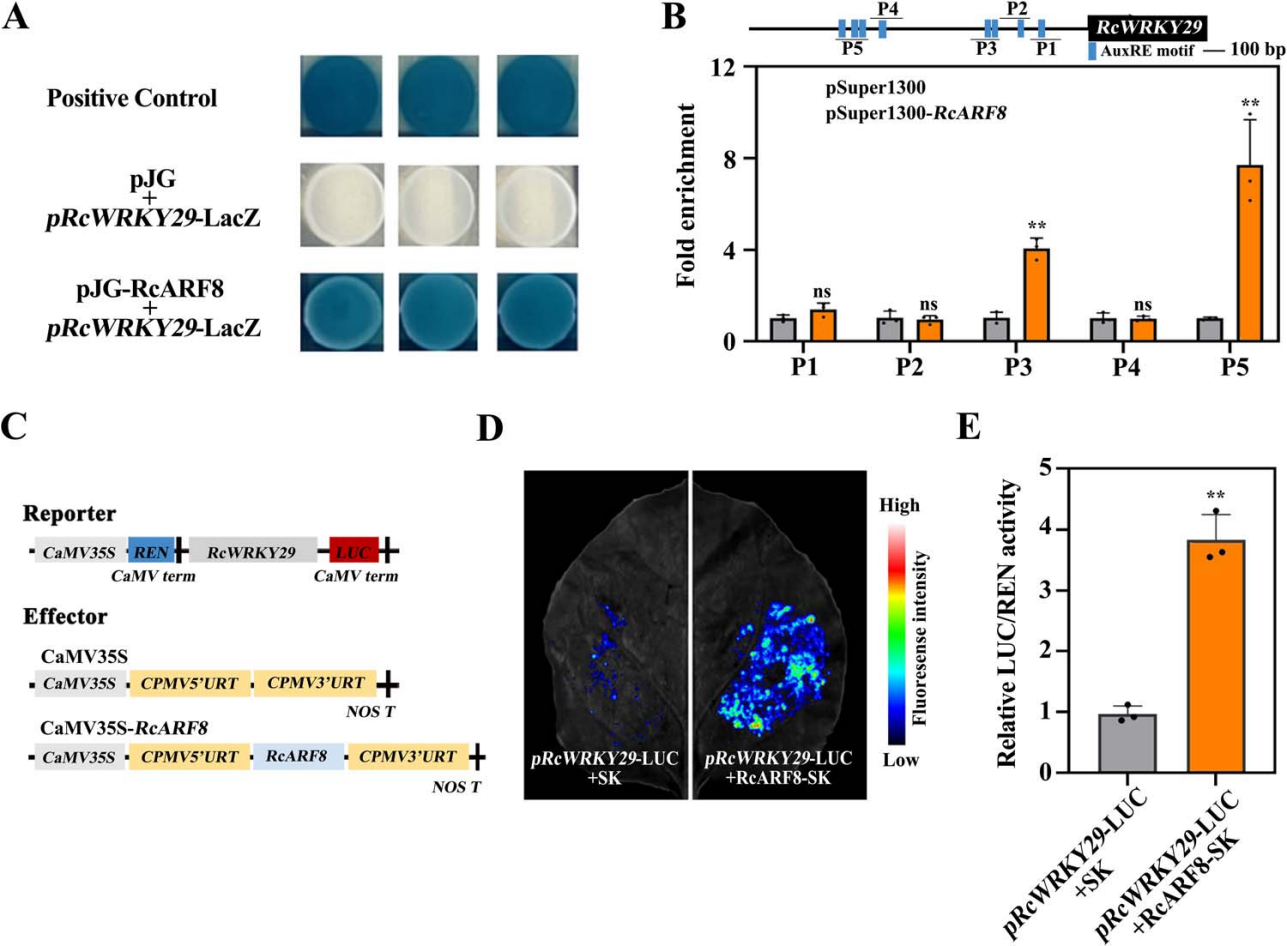
RcARF8 transactivates *RcWRKY29* promoter activity. (A) Y1H assay demonstrating the binding of RcARF8 to the promoter of *RcWRKY29*. Colony development is shown for the positive control, pJG + pRcWRKY29-LacZ, and pJG-*RcARF8* + *pRcWRKY29*-LacZ. (B) Chromatin immunoprecipitation (ChIP) analysis showing RcARF8 enrichment at different positions (P1-P5) on the *RcWRKY29* promoter. Values represent mean ± SD (*n* = 3). (C–E) Diagrams of the constructed vectors (C), Luciferase imaging of *N. benthamiana* leaves (D) and Quantification of the LUC/REN ratio (E). Values represent mean ± SD (*n* = 3). Significance was evaluated by two-tailed Student’s *t*-test (^**^P < 0.01, ^***^  *P* < 0.001, ns, not significant).

**Figure 8:**
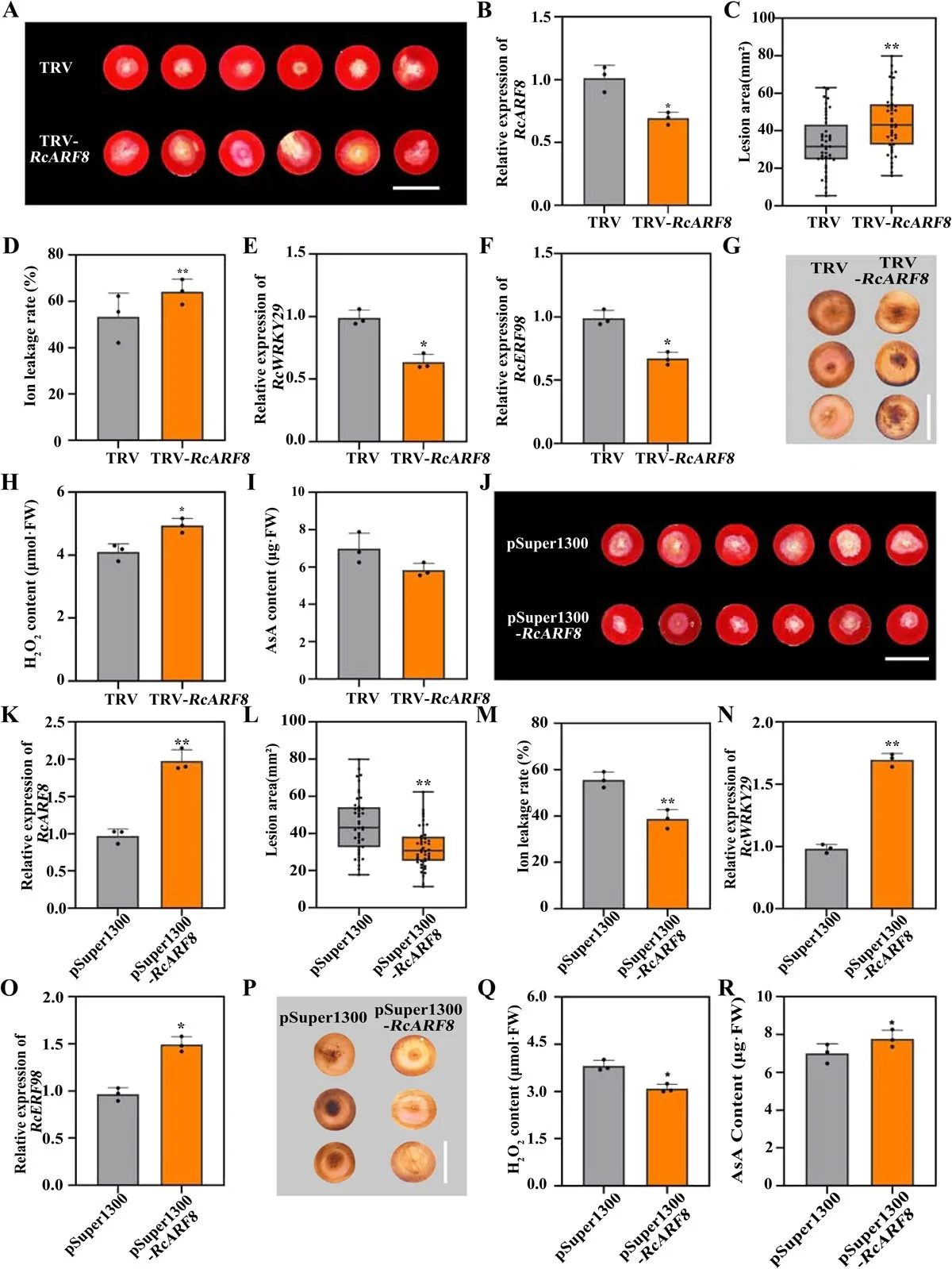
RcARF8 regulates resistance of rose petals against *B. cinerea* by controlling the RcWRKY29-RcERF98 module. (A) Lesion phenotypes of petal discs in TRV-*RcARF8* and TRV control. White scale bar = 1 cm. (B–D) Relative expression of *RcARF8* (B) (*n* = 3), lesion area (C) (*n* = 50), and relative ion leakage rate (D) (*n* = 3) of petal discs in TRV-*RcARF8* and TRV control. Values represent mean ± SD. (E, F) Relative expression of *RcWRKY29* and *RcERF98* after *RcARF8* silencing. Values represent mean ± SD (*n* = 3). (G) DAB staining of TRV-*RcARF8* and TRV of rose petals. White scale bar = 1 cm. (H and I) H_2_O_2_ and AsA levels of TRV-*RcARF8* and TRV control petals (*n* = 3). (J) Lesion phenotypes of petal discs in pSuper1300-*RcARF8* and pSuper1300 control. White scale bar = 1 cm. (K–M) Relative expression of *RcARF8* (K) (*n* = 3), lesion area (L) (*n* = 50) and relative ion leakage rate (M) (*n* = 3) of pSuper1300-*RcARF8* and pSuper1300 petals. Values represent mean ± SD. (N and O) Relative expression of *RcWRKY29* and *RcERF98* after *RcARF8* overexpression. Values represent mean ± SD (*n* = 3). (P )DAB staining of pSuper1300-*RcARF8* and pSuper1300 of rose petals. White scale bar = 1 cm. (Q and R) H_2_O_2_ and AsA levels of pSuper1300-RcARF8 and pSuper1300 petals (*n* = 3). Significance was evaluated by a two-tailed Student’s *t*-test (^*^  *P* < 0.05, ^**^  *P* < 0.01).

**Figure 9:**
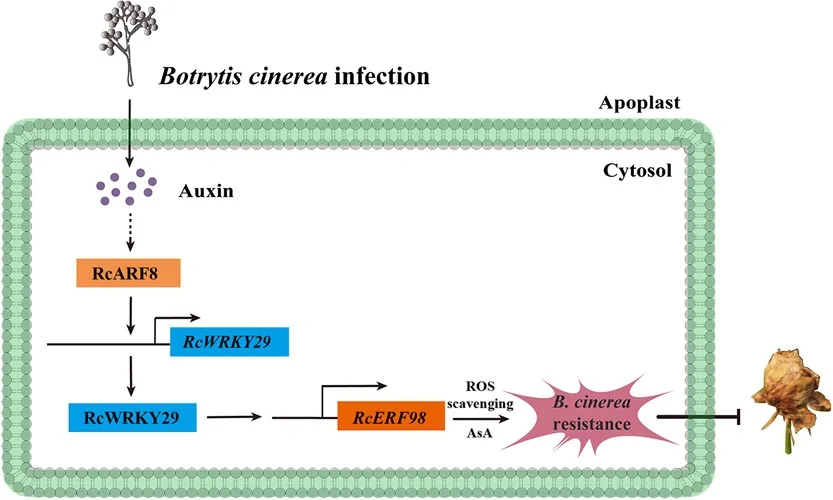
The proposed model: RcARF8- RcWRKY29 -RcERF98 module regulates the resistance to *B. cinerea* in rose petals. After *B. cinerea* infection in rose petals, endogenous auxin levels are elevated, which appears to induce *RcARF8* expression. The increased RcARF8 then binds to and activates the promoter of RcWRKY29. In turn, RcWRKY29 upregulates transcript levels of *RcERF98*, leading to enhanced AsA levels and ROS scavenging, and thereby potentially increasing defense against *B. cinerea* in rose petals.

## Discussion

In the flower industry, cut roses are highly susceptible to grey mold disease caused by *B. cinerea* during cultivation, leading to substantial economic losses. WRKY transcription factors play key roles in regulating plant growth and development, response to abiotic stresses, and defense against diseases. Previously, following the identification of 56 WRKY genes across the rose genome, transcriptomic data revealed a significant increase in the expression of this gene family in *R. hybrida* following infection by *B. cinerea* [16]. Studies have shown that the silencing of *RcWRKY13* confers enhanced resistance against *B. cinerea* in rose petals, whereas the silencing of *RcWRKY41* and *RcWRKY30* increases their disease susceptibility [16, 17, 20]. Here, we found that *RcWRKY29* expression was significantly induced by *B. cinerea* infection. Additionally, we found that there were no significant differences in *RcWRKY13*/*RcWRKY30/RcWRKY41* expression in *RcWRKY29*-silenced or *RcWRKY29-*overexpression petals (Supplementary Fig. S9).

WRKY transcription factors regulate plant defense gene expression during pathogen invasion, either by directly activating or repressing downstream target genes or through indirect feedback mechanisms. The enhanced resistance provided by RcWRKY13 is regulated by its influence on the expression of *RcCKX3* and *RcABI4* [17]. RcWRKY30 strengthened defense against *B. cinerea* by promoting the deposition of lignin in rose petals [20]. In our study, *RcWRKY29* was found to enhance resistance to *B. cinerea* infection in rose petals.

APETALA2-ETHYLENE RESPONSIVE FACTOR (AP2-ERF) family transcription factors are central regulators of immune responses across diverse crop species [41–43]. For example, *AtERF9* confers resistance to *B. cinerea* [44]. *In Arabidopsis, AtERF98* acts as a transcription regulator that enhances salt stress tolerance by activating AsA biosynthesis and improving the oxidative stress management [33]. Our previous study has revealed that *RcERF98* regulates reactive ROS scavenging under salt stress conditions [34]. In this study, *RcERF98* enhances resistance against *B. cinerea* in rose petals by regulating reactive AsA biosynthesis and ROS scavenging (Figs 4 and 5). WRKY and ERF transcription factors often form cascade regulatory relationships. For instance, in cotton, phosphorylated GhWRKY40a can directly activate *GhERF1b*, which subsequently induces the expression of defense genes and significantly enhances resistance to *Verticillium wilt* [45]. In our study, RcWRKY29 directly binds to and activates the promoter activity of *RcERF98*, thereby promoting the AsA biosynthesis and ROS scavenging, finally enhancing the resistance to *B. cinerea* infection in rose petals. Silencing or overexpressing *RcERF98* did not significantly change the expression level of *RcWRKY29* (Supplementary Fig. S10). These results suggested that ERF family members are involved in plant immune responses.

In this study, we observed that in rose petals, RcERF98 could regulate *RcVTC1* expression (Fig. 5E and F) and enhance resistance to *B. cinerea*, yet the accumulation level of AsA showed relatively limited changes. This limited increase in AsA suggests that AsA-dependent ROS scavenging alone is unlikely to fully account for the observed resistance, and that other downstream mechanisms may also contribute. The relatively small AsA change may be attributed to the fact that AsA, as a metabolic end product, maintains homeostasis through a dynamic balance between multi-step enzymatic reactions in its biosynthetic pathways (e.g. the D-mannose/L-galactose pathway) and catabolic processes [32]. Because VTC1 acts upstream in the AsA biosynthetic pathway, transcriptional alterations in VTC1 are likely buffered by downstream metabolic flux, enzymatic feedback regulation, or cellular transport mechanisms, resulting in relatively subtle fluctuations in AsA levels. Furthermore, the disease resistance mechanism mediated by RcERF98 likely operates through multiple coordinated pathways: beyond its potential role in modulating AsA biosynthesis, RcERF98 may also enhance immunity by directly regulating other defense-related genes, including pathogenesis-related genes and key antioxidant enzyme genes [46], thereby establishing a multi-layered immune response network that collectively strengthens rose resistance to *B cinerea*.

Although plant defense hormones such as SA, JA, ABA, and ET play crucial roles in plant immune responses [1], auxin also plays an important role in the response to pathogen invasions [10, 11]. A previous study reported significant increases in IAA levels in tomato leaves during *B. cinerea* pathogenesis [11]. In our study, we also found that the IAA level increased in response to *B. cinerea* (Fig. 6). Like in kiwifruit, exogenous NAA significantly increased the resistance of rose petals to *B. cinerea* [10]. However, the specific role of auxin signaling in plant–pathogen interactions is incomplete. Researchers have revealed that the activation of auxin signaling can increase plant resistance to certain necrotrophic pathogens and plant viruses [47, 48]. Conversely, during interactions with biotrophic or hemibiotrophic pathogens, auxin signaling appears to predominantly facilitate disease progression [8, 9]. For example, *OsARF17* increases disease resistance by interacting with different types of rice virus proteins [13]. In tomato, *StARF16* functions as a negative regulator of defense against necrotrophic pathogens by directly associating with the *StNPR1* promoter and suppressing its transcriptional activity [14]. In rose, RcARF8 regulates the RcWRKY29-RcERF98 module, thereby enhancing the AsA biosynthesis and ROS scavenging after *B. cinerea* infestation (Fig. 9).

We discovered that RcARF8 increases resistance to *B. cinerea* by controlling the RcWRKY29-RcERF98 pathway. This provides a new theoretical basis for hormone-mediated defence in roses and identifies a practical target for breeding. The module can be used as a molecular marker to identify favorable alleles (e.g. high-expression RcARF8 haplotypes) in germplasm, thereby accelerating the development of resistant varieties. As an alternative approach, CRISPR/Cas9 editing could be used to enhance *RcARF8* expression, or transgenic overexpression of *RcWRKY29* could be employed to directly improve resistance in susceptible cultivars. These findings offer clear strategies for translating our research into practical disease control applications.

## Materials and methods

### Plant materials and hormone treatments

The cut roses (*R. hybrida*) employed in the experiments were collected at stage 2 of development from the greenhouse facilities of the Flower Research Institute, Yunnan Academy of Agricultural Sciences [49]. We performed hormone treatments according to established methods [17]. Specifically, the rose petals of the outermost two whorls were punched into 10 mm discs, rinsed with ultrapure water, and transferred to 1% (w/v) agar plates containing 50 μM NAA, 30 μM NPA, 100 mM 6-BA, 100 mM ABA, and 50 μM MeJA, or in deionized water as a control for 24 h. The temperature was set at 25°C, with a 16-hour light/8-hour dark cycle and 60%–70% humidity. 16 discs were distributed per petri dish, which were subsequently used for *B. cinerea* inoculation.

### *B. cinerea* inoculation

*B. cinerea* was inoculated onto rose petal discs according to previously described methods [50]. The CAU8324 isolate of *B. cinerea* was maintained on potato dextrose agar (PDA) at 25°C for 3 weeks or more; conidia were harvested in sterile H_2_O, diluted to 1 × 10^5^ conidia/ml in half-strength PDB, and used immediately for inoculation. Two microlitres of *B. cinerea* suspension or PDB (control) were applied to the center of each petal disc for fungal inoculation, and Petri dishes were sealed to maintain humidity. Phenotypes were evaluated by measuring the lesion area at 72 hpi using a minimum of fifty petal discs per treatment with ImageJ software; significance was evaluated by two-tailed Student’s *t* test.

### VIGS and overexpression analyses

The VIGS experiment was performed according to previously described methods [11]. The specific fragments (250–350 bp) of *RcARF1*, *RcARF8*, *RcWRKY29*, and *RcERF98* were amplified via PCR and subsequently cloned and inserted into the pTRV2 vector. The recombinant pTRV2 and pTRV1 vectors were introduced into *Agrobacterium tumefaciens* strain GV3101. Transformants were grown for 18 h at 28°C with shaking (200 rpm) in LB containing kanamycin (25 μg/ml) and rifampicin (50 μg/ml). After harvesting, the cells were resuspended in infiltration buffer (10 mM MgCl_2_, 200 mM acetosyringone, and 10 mM MES, pH 5.6) with a final OD600 of approximately 1.0. An appropriate ratio of TRV1 to TRV2 agrobacterial suspensions was prepared, and rose petal discs were infiltrated under vacuum following the published procedure [17].

The CDS of *RcARF8*, *RcWRKY29*, and *RcERF98* were PCR-amplified and ligated into pSuper1300, yielding C-terminal fusion constructs. Both the resulting plasmids and the empty backbone were electroporated into *A. tumefaciens* strain GV3101, which was subsequently cultured in LB media supplemented with kanamycin (25 μg/ml) and rifampicin (50 μg/ml) at 28°C on a rotary shaker (200 rpm) for 18 hours. After harvesting, cells were pelleted and resuspended in infiltration medium to an optical density (OD600) of ~1.0. The bacterial suspensions were resuspended on a shaker for 1 hour (200 rpm, 28°C), and rose petal discs were vacuum infiltrated following the VIGS protocol. Six days after TRV and overexpression infection, rose petal discs were challenge-inoculated with B. *cinerea*; lesion diameters were measured at the designated time and analyzed by Student’s *t* -test.

### Subcellular localization

The full-length CDSs of *RcWRKY29*, *RcARF8*, and *RcERF98* were fused to *GFP* and inserted into the pSuper vector (pSuper::*RcWRKY29*, pSuper::*RcARF8,* and pSuper::*RcERF98*). Nuclei were visualized with DAPI counterstaining. Each recombinant construct was transformed into *A. tumefaciens* GV3101, and the resulting suspension was syringe-infiltrated into leaves of *Nicotiana benthamiana*. After three days, leaf samples were visualized with an Olympus FV3000 confocal laser-scanning microscope: GFP fluorescence was excited at 488 nm and DAPI was excited at 405 nm.

### RNA extraction and RT-qPCR

Total RNA was isolated from rose petals following the protocol [51]. Complementary DNA was reverse-transcribed from 1 μg of RNA with TransScript One-Step gDNA Removal and HiScript III RT SuperMix for qPCR (+gDNA wiper) (Vazyme) according to the supplier’s manual. RT-qPCR was performed on a StepOnePlus instrument (Applied Biosystems) using 2 × M5 Hiper SYBR Premix EsTaq (with Tli RNase) (Mei5bio) under conditions specified by the manufacturer. Transcript levels were normalized against the reference gene RcUBI2. The primers are provided in Supplementary Table S2.

### RNA sequencing

To investigate the transcriptional dynamics during fungal infection, rose petals were harvested at 0, 24, 36, and 48 hpi with *B. cinerea*. Three biological replicates were collected at each time point. All samples were flash-frozen in liquid nitrogen and stored at −80°C for RNA sequencing. Following the manufacturer’s instructions, cDNA libraries were constructed and then subjected to paired-end sequencing on an Illumina NovaSeq 6000 platform. Raw reads were filtered with Trimmomatic, aligned to the *R. chinensis* ‘Old Blush’ reference genome (NCBI BioProject 8 255 808), and assembled with StringTie. Transcript abundance was estimated as FPKM and differential expression was declared for genes exhibiting |log_2_fold-change| ≥ 1 and FDR-adjusted *q* ≤ 0.05. Significant genes were functionally annotated and mapped to KEGG pathways. All treatments were represented by three independent biological replicates, and the cleaned reads have been deposited in NCBI BioProject PRJNA1202213.

### Quantification of endogenous IAA in rose petals

Endogenous IAA was quantified in 100 mg fresh-weight petal tissue following the protocol [35]. Samples were snap-frozen in liquid nitrogen and kept at −70°C until analysis. IAA levels were measured by MetWare (Wuhan, China) via the AB Sciex QTRAP 6500 LC–MS/MS platform (UPLC, ExionLC AD, MS, Applied Biosystems 6500; Triple Quadrupole, https://sciex.com.cn/). Each sample included three biological replicates.

### DAP-seq of RcWRKY29

DAP-seq experiments were performed according to published methods [52]. Genomic DNA was extracted from rose petals and used to construct an affinity purification library with the MICH TLX DNA-Seq Kit (MICH, NGS0602). A Halo-tagged RcWRKY29 protein expression vector was generated and expressed via a wheat germ in vitro translation system. The Halo-tagged RcWRKY29 protein was incubated with the DNA library, and DNA fragments bound to RcWRKY29 were enriched via magnetic beads. Nonspecific chromatin was removed through multiple washing steps. The enriched DNA-protein complexes were purified, and the associated DNA fragments were sequenced via paired-end 150 bp (PE150) sequencing on an Illumina NovaSeq 6000 platform. The sequencing reads were mapped to the reference genome (*R. chinensis* ‘Old Blush,’ GenBank ID 8255808) via BWA-MEM [53]. Peak calling and genome-wide binding site analysis were performed via MACS [54]. The DAP-seq reads were deposited in the NCBI BioProject database under accession number PRJNA1464212.

### Y1H assay

Promoter fragments of *RcWRKY29* and *RcERF98* (2000 bp) were subsequently cloned and inserted into the pLacZ vector to generate *pRcWRKY29*-LacZ and *pRcERF98*-LacZ. The CDSs of *RcARF1*, *RcWRKY29,* and *RcARF8* were subsequently cloned and inserted into the pJG4–5 vector to create pJG-RcARF1, pJG-RcWRKY29, and pJG-RcARF8. Each pJG effector construct was co-introduced with the corresponding pLacZ reporter into Saccharomyces cerevisiae EGY48 by the lithium-acetate method. Transformants were first selected on synthetic dextrose (SD) medium -Trp and -Ura, then replica-plated onto SD plates containing 80 μg/ml X-gal for β-galactosidase colorimetric detection [17].

### Dual-luciferase reporter assay

For the evaluation of the transcriptional activity of *RcARF1*, *RcARF8* and *RcWRKY29*, a dual-luciferase reporter assay was performed. A reporter cassette carrying five tandem GAL4 sites upstream of the minimal CaMV 35S promoter was fused to firefly luciferase (LUC), while CaMV 35S-driven Renilla luciferase (REN) provided the internal reference. The open reading frames of *RcARF1*, *RcARF8*, and *RcWRKY29* were cloned and inserted into the pGreenII62-SK vector, while the promoters of *RcWRKY29* and *RcERF98* were cloned and inserted into the pGreenII0800-LUC dual-reporter vector [55]. Empty vectors were used as controls. The effector and reporter constructs were mobilized into *Agrobacterium* strain GV3101 that additionally harbored the helper *pSoup* plasmid. Equal volumes of *Agrobacterium* cultures containing the constructs were mixed and pressure-infiltrated into *N. benthamiana* leaves. Three days after infiltration, leaf discs were lysed and LUC/REN ratios were determined with the Dual-Luciferase Reporter Assay System (Promega) on a Microplate Reader; spatial luminescence was captured with a CCD camera. Primer sequences are listed in Supplementary Table S2.

### ChIP quantitative PCR

ChIP quantitative PCR (ChIP–qPCR) was executed according to the reported methods [35]. Petal tissues that had transiently expressed GFP-fusion constructs were fixed with 1% (w/v) formaldehyde, after which the cross-linking reaction was terminated by supplementing the medium with glycine to 0.125 M. The crosslinked chromatin was fragmented via sonication to produce DNA fragments ranging from 300 to 750 bp. GFP-Trap® A (1:1000 dilution; Chromotek, gtma-20) was used to immunoprecipitate the GFP-tagged protein–DNA complexes. After overnight incubation at 4°C with the antibody, magnetic beads were used to isolate the bound complexes. The beads were thoroughly washed, and the DNA was eluted. The crosslinks were then reversed by incubation at 65°C for 6 h, and the DNA was purified via a PCR purification kit. The resulting DNA was analyzed by qPCR with specific primers designed to amplify different regions. The primers used for qPCR are listed in Supplementary Table S2.

### DAB staining

Using the 3,3′-diaminobenzidine (DAB) staining method for *in situ* H₂O₂ localization. Petals were immersed in DAB staining solution (pH 3.8), vacuum-infiltrated, and incubated in the dark for 10 hours. The stained petals were then destained in destaining solution (glycerol/acetic acid/ ethanol [1:1:3, v/v/v]) for 72 hours before photographic documentation of the staining patterns.

### Ion leakage measurements

Uniform rose leaves were selected and thoroughly rinsed with distilled water. The leaves were placed into a 50 ml centrifuge tube, followed by the addition of 40 ml of distilled water. A vacuum pump was employed to apply suction at 0.1 MPa for 5 minutes, after which the tube was shaken at 220 rpm for 1 hour and allowed to stand for an additional minute. The conductivity of the solution in the tube at this stage was measured using a conductivity meter and recorded as R1. Subsequently, the tube was boiled in water for 10 minutes, cooled to room temperature, and the conductivity of the solution was measured again, recorded as R2. The electrolyte leakage rate was calculated using the formula (R1/R2) × 100%.

### H_2_O_2_ and AsA content measurements

Hydrogen peroxide was quantified with the Solarbio Hydrogen Peroxide Assay Kit. Petal tissue (0.1 g) was ground in 1 ml ice-cold extraction buffer, centrifuged at 8000 × g for 20 min at 4°C, and the supernatant was retained. After addition of the kit reagents, the reaction mixture was incubated for 5 min and the absorbance was read at 415 nm.

For ascorbate (AsA), 0.1 g tissue was processed identically, and the supernatant was assayed with the Solarbio AsA detection kit according to the manufacturer’s protocol; absorbance was recorded at 420 nm.

### Phylogenetic analysis

Protein sequences were first aligned with DNAMAN, after which MEGA-X was employed to infer evolutionary relationships; trees were generated under the maximum-likelihood criterion. This analysis incorporated 1000 bootstrap replicates and utilized the Jones–Taylor–Thornton substitution model.

### Statistical analysis

Every assay was performed with at least three independent biological replicates. Pairwise comparisons were evaluated by two-tailed Student’s *t*-test, whereas multiple-group comparisons were assessed by one-way ANOVA followed by Duncan’s multiple-range test; differences were deemed significant at *P* < 0.05.

## Supplementary Material

Web_Material_uhag224

## Data Availability

All data supporting the findings of this study are available within the paper and within its supplementary data published online. The transcriptome and DAP-seq data were deposited in the NCBI Sequence Read Archive database under accession numbers PRJNA1202213 and PRJNA1464212.
