# Supplementary material for: Auxin-mediated RcARF8-RcWRKY29-RcERF98 module protects the rose petals against the Botrytis cinerea by modulating ascorbic acid biosynthesis and reactive oxygen species scavenging
Source: Hortic Res. 2026 Jun 3;13(11):uhag224. doi: 10.1093/hr/uhag224 (PMC13722868; doi:10.1093/hr/uhag224)
Supplement: Supplementary file 1 [file web_material_uhag224.zip › Supplementary figure.pdf]

Auxin-mediated RcARF8-RcWRKY29-RcERF98 module protect the rose petals against the *Botrytis cinerea* by modulating AsA biosynthesis and ROS scavenging

Jie Wu[1,3+], Mengyue Jing[1+], Shiqiu Yang[1,2+], Qiushi Li[1,2], Qi Fu[1,2], Xujun Ma[1,2], Dan Wang[1,2], Jiaxing Li[1], Hongying Jian[1], Huichun Wang[1], Huijun Yan[1], Kaixue Tang[1], Hao Zhang[1*], Xianqin Qiu[1*], Weikun Jing[1*]

[1] *Flower Research Institute of Yunnan Academy of Agricultural Sciences, Kunming, Yunnan, 650205, China*

[2] *Institute of Plant Resources, Yunnan University, Kunming Yunnan, 650091, China*

[3] *Vegetables and Flowers Institute of Inner Mongolia Academy of Agricultural & Animal Husbandry Sciences, Hohhot, 010031, China*

[+]These authors contributed equally to this work (J.W., M.J., and S.Y.)

*Author for correspondence:

Weikun Jing (jwk093@163.com; ORCIDs: 0000-0001-7177-7560), Xianqin Qiu (532461500@qq.com) and Hao Zhang (zh13987187107@163.com)

Supplementry Figures

Supplementary Figure 1 Characterization of RcWRKY29.

Supplementary Figure 2 Y1H analysis of the binding of RcWRKY29 to the candidate gene promoter.

Supplementary Figure 3 Characterization of RcERF98.

Supplementary Figure 4 Relative expression levels of AsA- and ROS-related genes.

Supplementary Figure 5 RcARF1 did not affect the transcriptional activity of *RcWRKY29* promoter.

Supplementary Figure 6 Functional analysis of *RcARF1* in rose petals against *Botrytis cinerea.*

Supplementary Figure 7 Relative expression of *RhWRKY29*, *RhERF98*, and *RhARF8* of NPA treatment and control petals.

Supplementary Figure 8 Protein-protein interactions among RcARF8, RcWRKY29, and RcERF98 using BiFC and Y2H assays.

Supplementary Figure 9 Relative expression of *RcWRKY13/30/41* in TRV- *RcWRKY29* and TRV petals.

Supplementary Figure 10 Relative expression of *RcWRKY29* in TRV- *RcERF98,* pSuper1300-*RcERF98* and control petals.

Supplementary Figure 11 KEGG enrichment analysis of RcWRKY29 target genes.

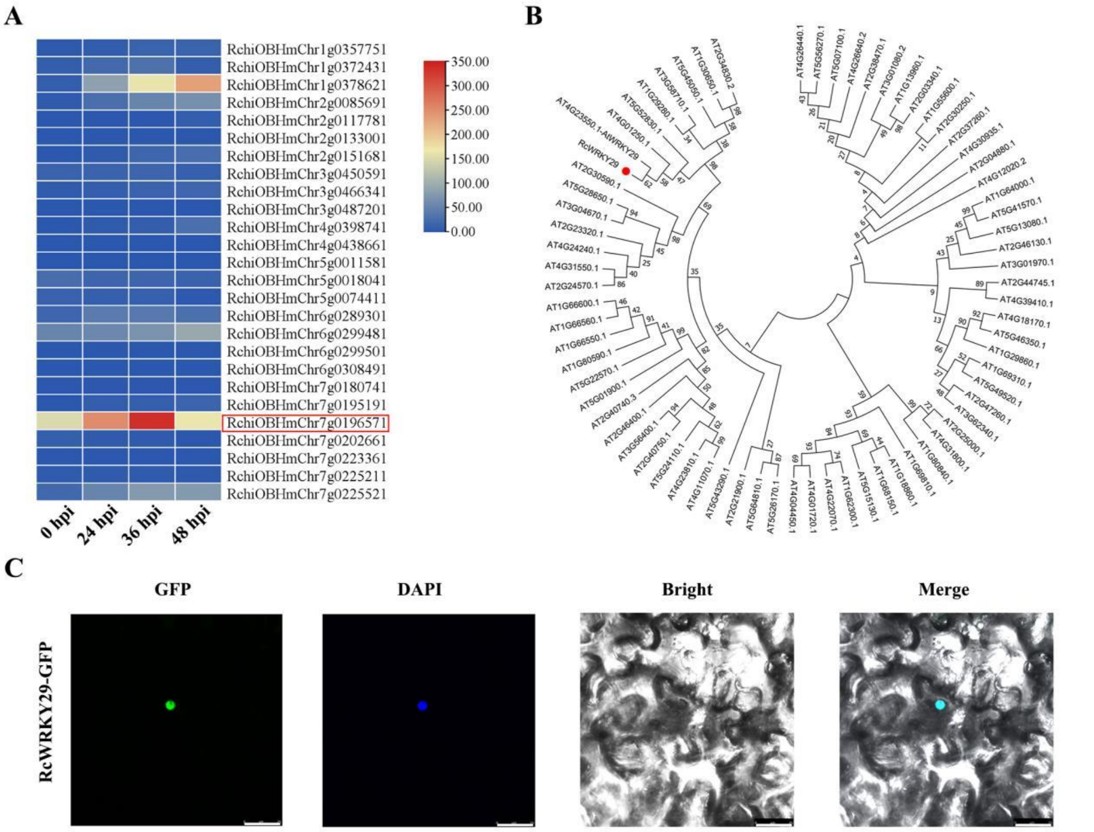

Supplementary Figure 1 Characterization of *RcWRKY29*.

A. Heatmap showing the expression patterns of *WRKY* genes among the differentially expressed genes (DEGs) at 0, 24, 36, and 48 hpi.

B. Phylogenetic analysis of *RcWRKY29* with Arabidopsis WRKY family proteins. *RcWRKY29* is highlighted with a red dot. The tree was constructed using the neighbor-joining method with 1,000 bootstrap replicates.

C. Subcellular localization of *RcWRKY29* in *N. benthamiana* leaves. *RcWRKY29*-GFP signals were expressed in *N. benthamiana* leaves. Fluorescence signal was visualized by confocal microscopy 3 days after infiltration. and DAPI staining represents the nucleus location. Scale bar, 25 μm.

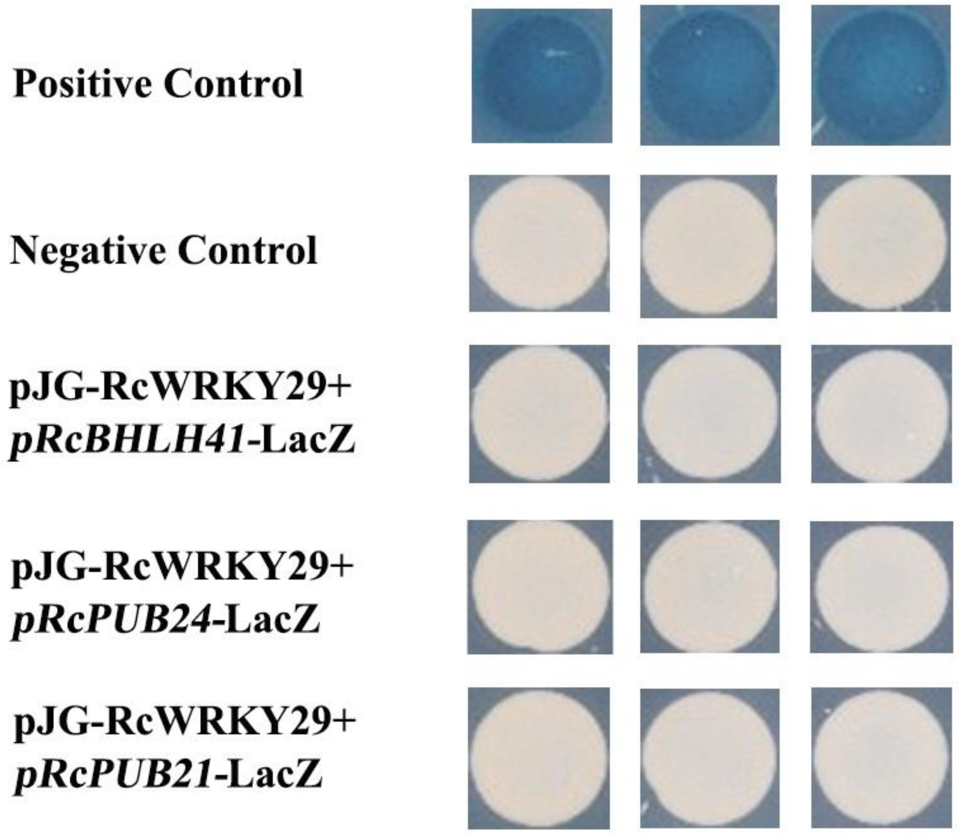

Supplementary Figure 2 Y1H analysis of the binding of RcWRKY29 to the candidate gene promoter.

Note, RcWRKY29 was tested for binding to *pRcBHLH141*-LacZ, *pRcPUB24*-LacZ, and *pRcPUB21*-LacZ. No binding interactions were observed under the experimental conditions.

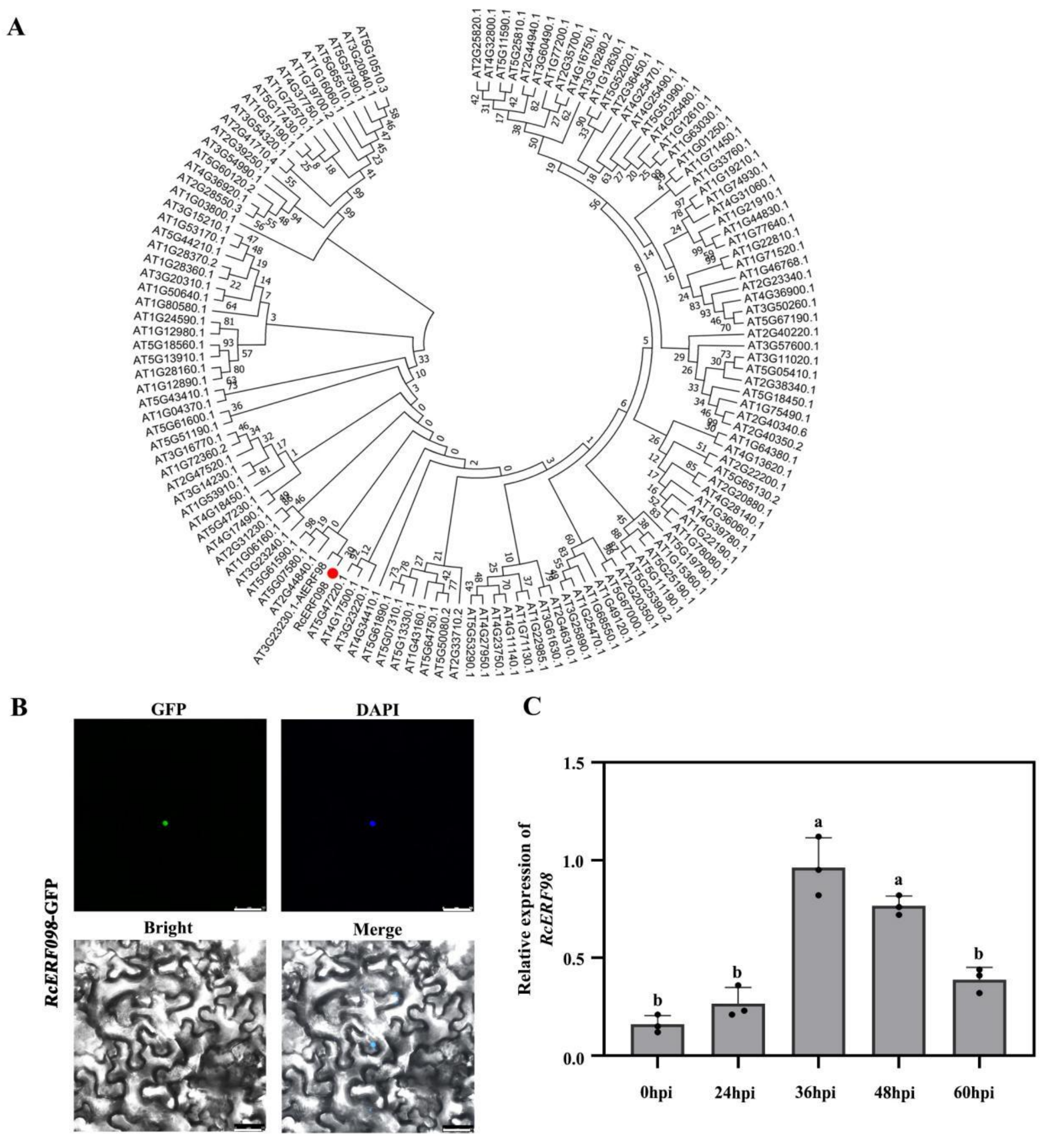

Supplementary Figure 3 Characterization of *RcERF98*.

A. Phylogenetic analysis of *RcERF98* with Arabidopsis ERF98 family proteins. The phylogenetic tree was constructed using the neighbor-joining method in MEGA 7.0 with 1,000 bootstrap replicates. *RcERF98* is highlighted with a red dot.

B. Subcellular localization of *RcERF98* in *N. benthamiana* leaves. *RcERF98*-GFP signals were expressed in *N. benthamiana* leaves. Fluorescence signal was visualized by confocal microscopy 3 days after infiltration. and DAPI staining represents the nucleus location. Scale bar, 50 μm.

C. Relative expression of *RcERF98* at different time points after inoculation (0, 24, 36, 48, 60 hpi). Data are means ± SD from three biological replicates.

Different lowercase letters (in A) indicate significant differences according to one-way ANOVA with Duncan's multiple comparisons test ($P < 0.05$).

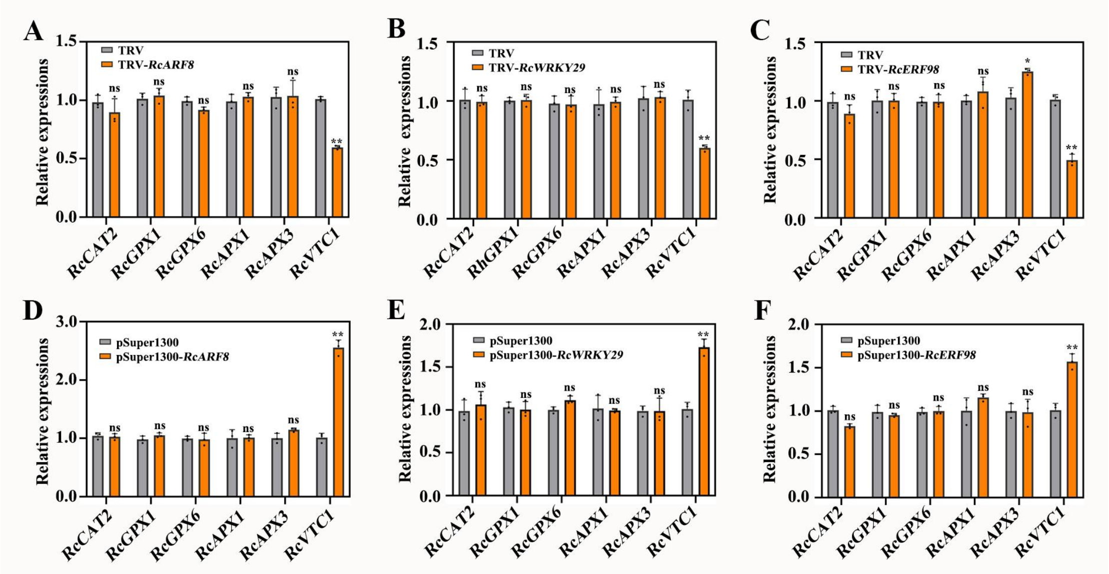

Supplementary Figure 4 Relative expression levels of AsA- and ROS-related genes in TRV-RcARF8 (A), TRV- RcWRKY29 (B), TRV- RcERF98 (C), pSuper1300-RcARF8 (D), pSuper1300-RcWRKY29 (E), pSuper1300-RcERF98 (F), and control petals.

Values represent mean ± SD (n =3). Significance was evaluated by two-tailed Student's t-test (* $P < 0.05$, ** $P < 0.01$, ns, not significant).

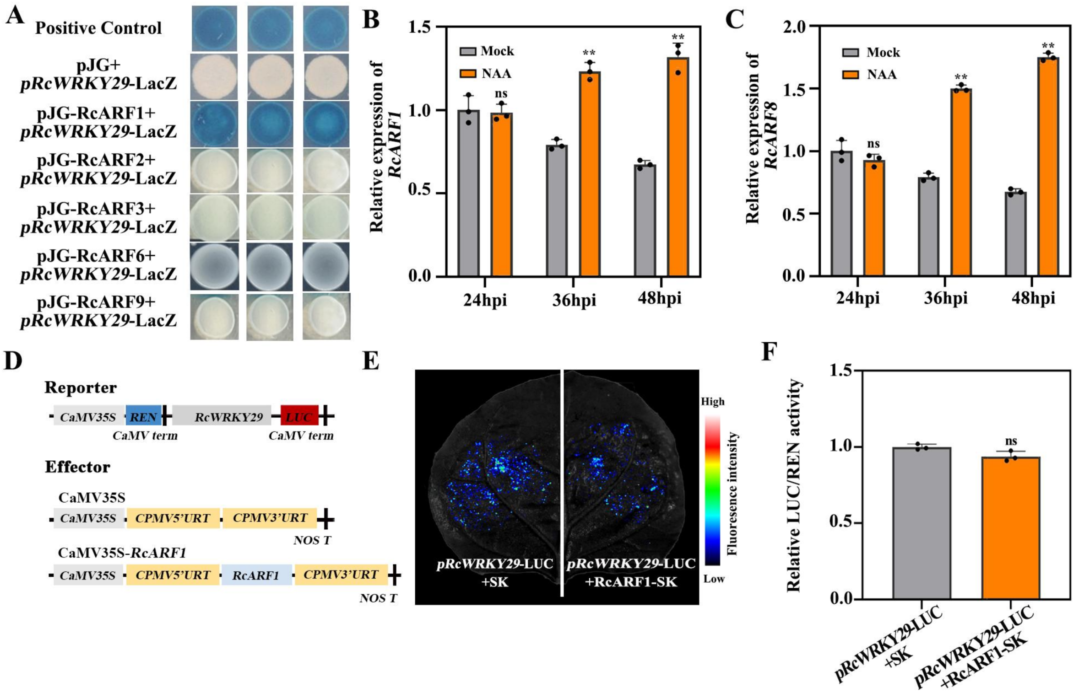

Supplementary Figure 5 RcARF1 did not affect the transcriptional activity of *RcWRKY29* promoter.

A. Yeast one-hybrid analysisof the binding of RcARFs to *RcWRKY29* promoter. As indicated, RcARF1 showed a significant interaction with *RcWRKY29* promoter, while RcARF2, RcARF3, RcARF6, and RcARF9 showed no interaction under the experimental conditions.

B-C. Expression levels of *RcARF1* and *RcARF8* in response to NAA treatment at different time points (24, 36, and 48 hpi). Data are means ± SD from three biological replicates.

D-F. Schematic representation of reporter and effector constructs used in the dual-luciferase reporter assay. The *RcWRKY29* promoter drives the firefly luciferase (LUC) gene, and Renilla luciferase (REN) serves as an internal control. The effector constructs contain RcARF1 under the control of the *CaMV35S* promoter (D); Representative fluorescence imaging in *N. benthamiana* leaves co-infiltrated with *pRcWRKY29*-LUC and the indicated effectors (E); Relative LUC/REN activity of the *RcWRKY29* promoter co-infiltrated with RcARF1 effector constructs (F).Data are means ± SD from three biological replicates.

Significance was evaluated by two-tailed Student's t-test (* $P < 0.05$, ** $P < 0.01$, ns, not significant).

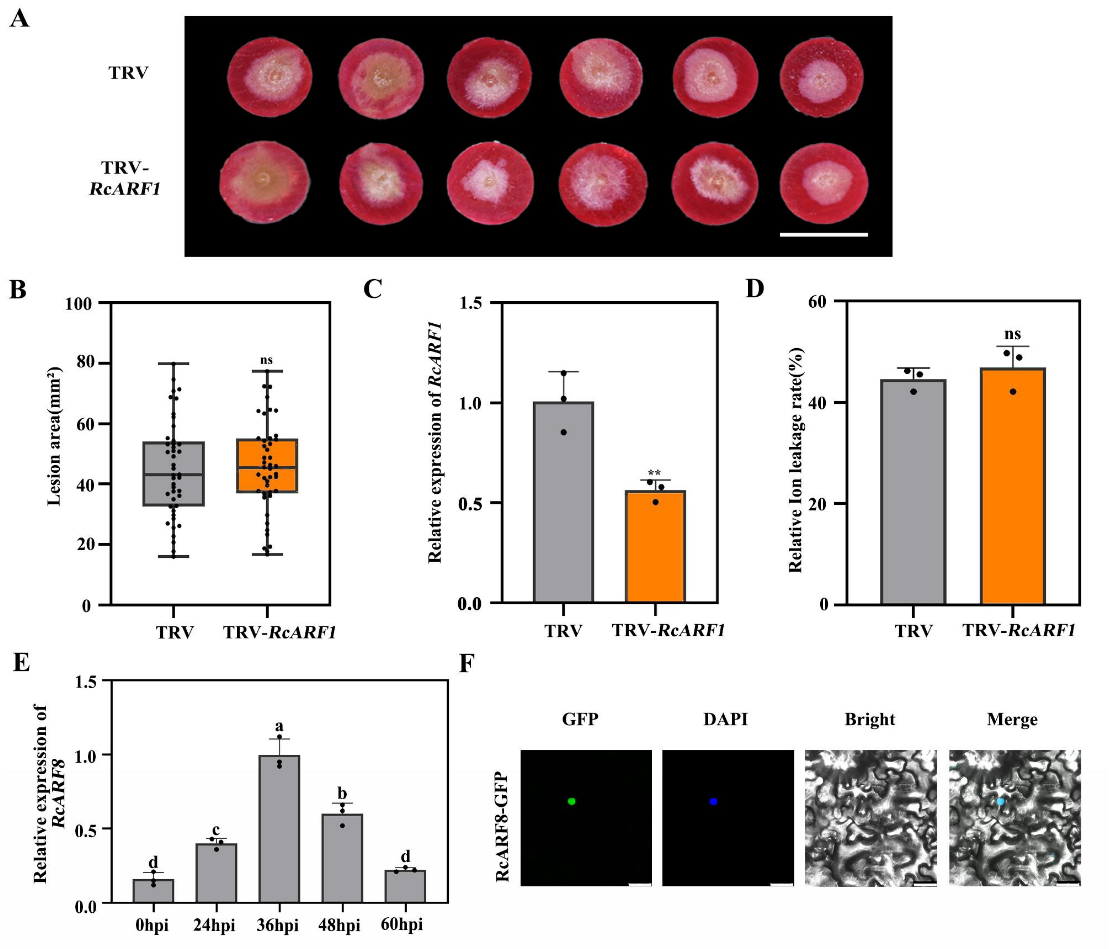

Supplementary Figure 6 Functional analysis of *RcARF1* in rose petals against *Botrytis cinerea.*

A. Representative images of lesion areas in rose petal discs infiltrated with TRV control and TRV-*RcARF1* constructs following infection with *B. cinerea*. White scale bar = 1cm.

B. Statistical analysis of lesion areas in TRV and TRV-*RcARF1* infiltrated petals. Data are presented as boxplots (n=50).

C. Relative expression of *RcARF1* in TRV and TRV-*RcARF1* petals. *RcARF1* expression was significantly reduced in TRV-*RcARF1* petals. Data are means ± SD from three biological replicates.

D. Relative ion leakage rate in TRV and TRV-*RcARF1* petals following *B. cinerea* infection. No significant differences were observed. Data are means ± SD from three biological replicates.

E. Relative expression of *RcARF8* at different time points after inoculation (0, 24, 36, 48, 60 hpi). Data are means ± SD from three biological replicates.

F. Subcellular localization of *RcARF8* in *N. benthamiana* leaves. *RcARF8*-GFP signals were

expressed in *N. benthamiana* leaves. Fluorescence signal was visualized by confocal microscopy 3 days after infiltration. and DAPI staining represents the nucleus location. Scale bar, 50 μm. Different lowercase letters (in E) indicate significant differences according to one-way ANOVA with Duncan's multiple comparisons test ($P < 0.05$).

Significance was evaluated by two-tailed Student's t-test (* $P < 0.05$, ** $P < 0.01$, ns, not significant).

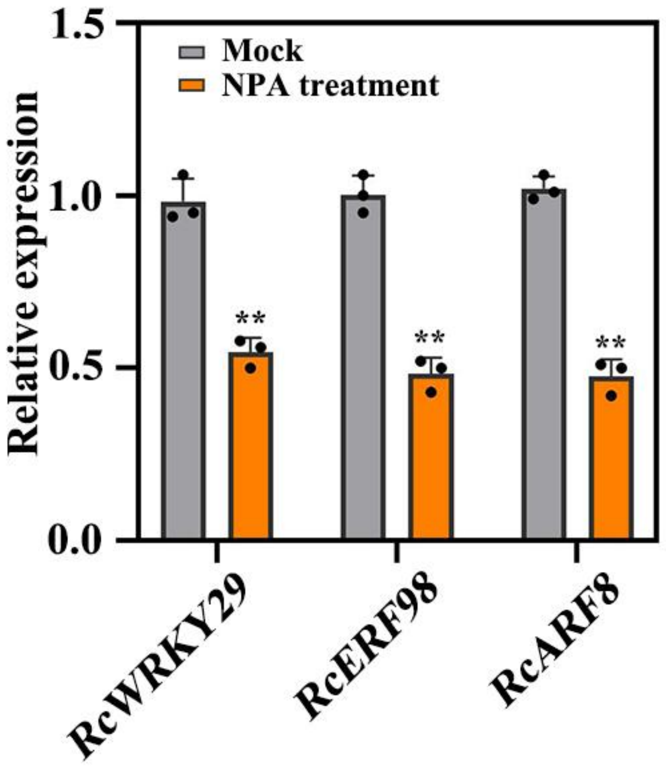

Supplementary Figure 7 Relative expression of *RhWRKY29*, *RhERF98*, and *RhARF8* of NPA treatment and control petals.

Significance was evaluated by two-tailed Student's *t*-test (* $P < 0.05$, ** $P < 0.01$, *** $P < 0.001$).

Data are means ± SD from three biological replicates.

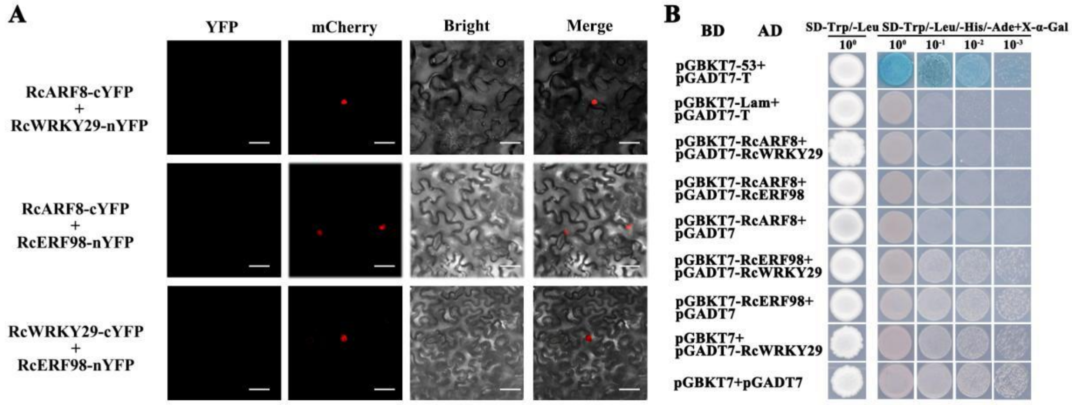

Supplementary Figure 8 Protein-protein interactions among RcARF8, RcWRKY29, and RcERF98 using BiFC (A) and Y2H assays (B).

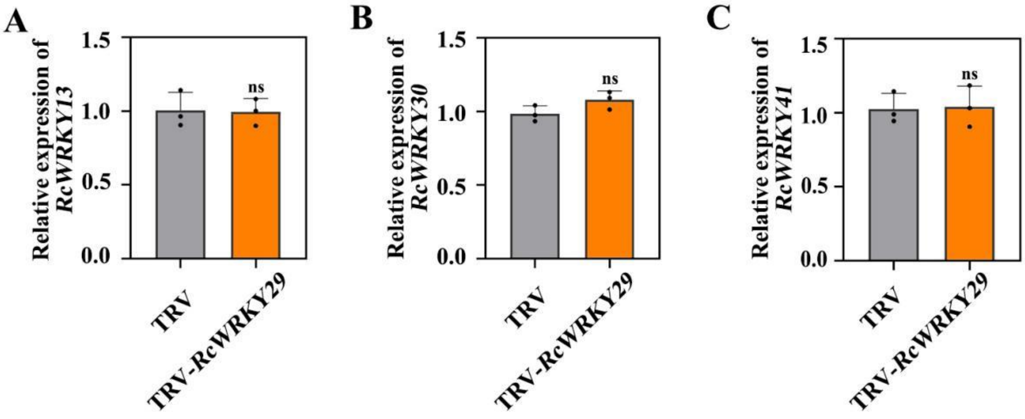

Supplementary Figure 9 Relative expression of *RcWRKY13/30/41* in TRV- *RcWRKY29* and TRV petals (n =3).

Significance was evaluated by two-tailed Student's t-test (* P < 0.05, ** P < 0.01, ns, not significant).

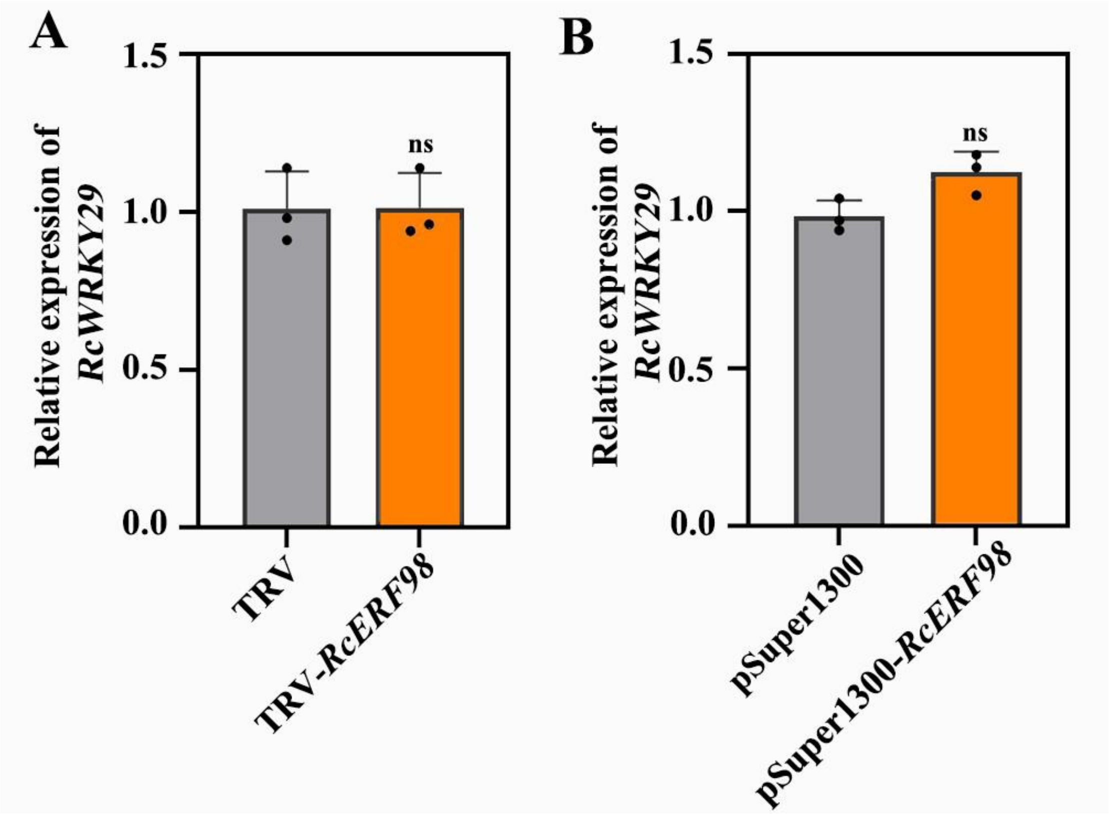

Supplementary Figure 10 Relative expression of *RcWRKY29* in TRV-*RcERF98,* pSuper1300-RcERF98 and control petals.

Significance was evaluated by two-tailed Student's t-test (* P < 0.05, ** P < 0.01, ns, not significant).

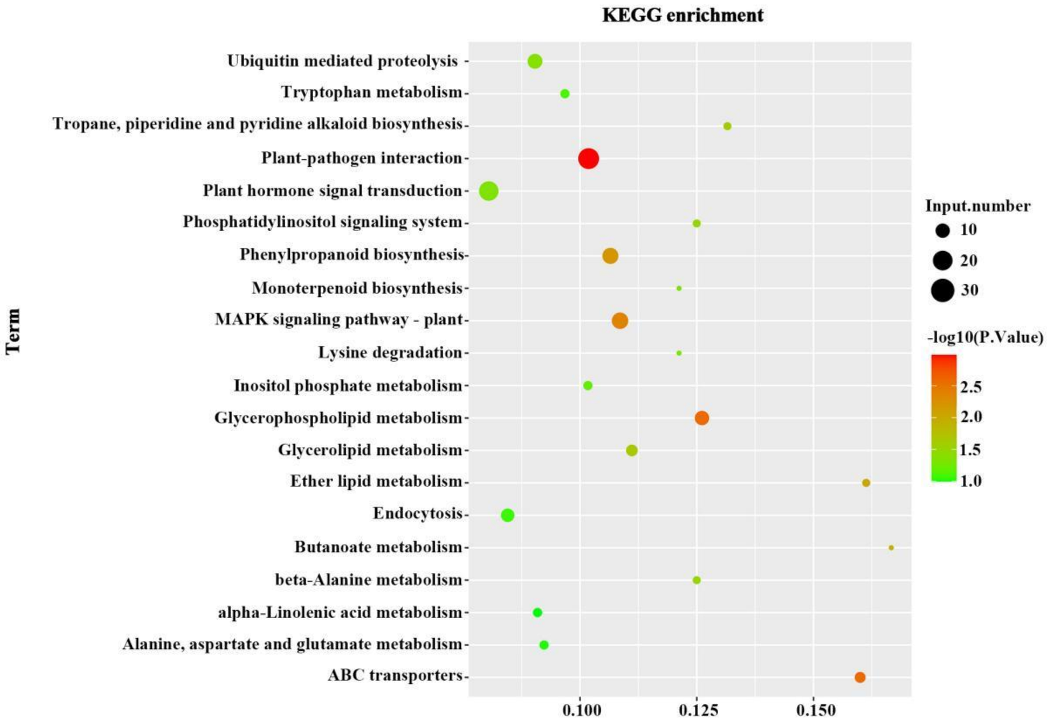

Supplementary Figure 11 KEGG enrichment analysis of RcWRKY29 target genes.
